# Identification and support of autistic individuals within the UK Criminal Justice System: a practical approach based upon professional consensus with input from lived experience

**DOI:** 10.1186/s12916-024-03320-3

**Published:** 2024-04-12

**Authors:** Emma Woodhouse, Jack Hollingdale, Lisa Davies, Zainab Al-Attar, Susan Young, Luke P. Vinter, Kwaku Agyemang, Carla Bartlett, Colleen Berryessa, Eddie Chaplin, Quinton Deeley, Ian Freckelton, Felicity Gerry, Gisli Gudjonsson, Katie Maras, Michelle Mattison, Jane McCarthy, Richard Mills, Peter Misch, David Murphy, Clare Allely

**Affiliations:** 1 Compass Psychology Services Ltd, London, UK; 2 Institute of Psychiatry, Psychology and Neuroscience, King’s College London, London, UK; 3 Expert Psychological Services, Brighton, UK; 4 University of Oxford, London, UK; 5 University College London, London, UK; 6 Victoria University, Melbourne, Australia; 7 University of Central Lancashire, Preston, UK; 8 Psychology Services Limited, London, UK; 9 University of Reykjavík, Reykjavík, Iceland; 10 Department of Criminology, University of Derby, Derby, UK; 11 Barnet, Enfield & Haringey NHS Trust, London, UK; 12 Tully Forensic Psychology Ltd, Nottingham, UK; 13 School of Criminal Justice, Rutgers University, Newark, NJ USA; 14 London South Bank University, Institute of Health and Social Care, London, UK; 15 National Autism Unit, South London and Maudsley NHS Foundation Trust, London, UK; 16 Law Faculty and Department of Psychiatry, University of Melbourne, Melbourne, Australia; 17 Castan Chambers, Melbourne, Australia; 18 Libertas Chambers, London, UK; 19 Crockett Chambers, Melbourne, Australia; 20 University of Bath, Bath, UK; 21 University of Chester, Chester, UK; 22 University of Auckland, Auckland, New Zealand; 23 AT-Autism, London, UK; 24 Department of Psychology, University of Bath, Bath, UK; 25 Royal College of Psychiatrists, London, UK; 26 Broadmoor Hospital, West London NHS Trust, London, UK; 27 School of Health and Society, University of Salford, Manchester, UK

**Keywords:** Autism, Forensics, Offending, Criminal justice system (CJS), Risk, Crime, Support, Assessment

## Abstract

**Background:**

Autism spectrum disorder (hereafter referred to as autism) is characterised by difficulties with (i) social communication, social interaction, and (ii) restricted and repetitive interests and behaviours. Estimates of autism prevalence within the criminal justice system (CJS) vary considerably, but there is evidence to suggest that the condition can be missed or misidentified within this population. Autism has implications for an individual’s journey through the CJS, from police questioning and engagement in court proceedings through to risk assessment, formulation, therapeutic approaches, engagement with support services, and long-term social and legal outcomes.

**Methods:**

This consensus based on professional opinion with input from lived experience aims to provide general principles for consideration by United Kingdom (UK) CJS personnel when working with autistic individuals, focusing on autistic offenders and those suspected of offences. Principles may be transferable to countries beyond the UK. Multidisciplinary professionals and two service users were approached for their input to address the effective identification and support strategies for autistic individuals within the CJS.

**Results:**

The authors provide a consensus statement including recommendations on the general principles of effective identification, and support strategies for autistic individuals across different levels of the CJS.

**Conclusion:**

Greater attention needs to be given to this population as they navigate the CJS.

## Background

This consensus statement addresses the identification and support strategies for autistic individuals within the criminal justice system (CJS), focusing on autistic offenders and those suspected of offences. The authors are mindful of the importance of language and the debates around terminology within autistic communities. Research suggests that there is an overall preference for ‘identity first’ language within autistic communities [1–3] so for the purposes of this paper we will use ‘autistic individuals’. However, we are aware that this may not reflect individual preferences.

We begin with a review of relevant literature as background to best practice in the identification and support for autistic individuals within the UK CJS.

### Autism

In the International Classification of Diseases, 11th revision (ICD-11) autism is characterised by, ‘Persistent deficits in initiating and sustaining social communication and reciprocal social interactions; Persistent restricted, repetitive, and inflexible patterns of behaviour, interests, or activities; Lifelong excessive and persistent hypersensitivity or hyposensitivity to sensory stimuli or unusual interest in sensory stimuli’ [4] and similarly described by the Diagnostic and Statistical Manual, 5th Edition Text Revision (DSM-5 TR) [5]. Due to diversity in presenting behaviours, autism is a highly heterogenous condition [6]. Estimated prevalence of autism in the general population is between 1 and 2.5% for children and adults [7, 8] and continues to increase as a result of awareness, earlier diagnoses and changes in diagnostic criteria [9, 10]. Reported diagnosis rates are three times higher in biological males than females [10, 11] but the biological male:female ratio is debated due to potential sex differences in detection, referral and assessment. It should be noted that the prevalence rates of autism for those who self-report gender incongruence is much higher. A recent meta-analysis utilising data from 25 studies consisting of 8662 participants identified an autism prevalence rate of 11% in individuals with gender dysphoria/incongruence [12]. Although the core features of autism are reported to remain stable across the lifespan [13], this is not universally accepted [14].

### Autism and the legal system

Despite the extensive literature on autism, research exploring autism within the CJS is limited [15, 16]. Barriers to completing high-quality research within the CJS include challenges in obtaining detailed developmental histories necessary for a comprehensive assessment, co-occurring conditions and differential diagnoses, and access to clinical populations in secure settings. Additional ethical considerations arise when attempting to complete research with individuals in secure settings. In response to historical abuse, the UK research regulatory bodies (such as the National Research Ethics Service) focus on protecting those in secure settings as vulnerable participants who have limited capacity for informed consent and whose confidentiality and privacy may not be assured, all whilst being within a restrictive or coercive environment [17]. These factors lead to small sample sizes and varied methodologies resulting in mixed research findings and conclusions that may be limited.

The core features of autism are not inherent risk factors for engaging in offending. Some literature reviews and meta-analyses consisting of a small number of studies indicate that autistic individuals are no more likely to engage in potential criminal offences than the general population [18–21]. Some research indicates that autistic individuals are less likely to engage in offending. For example, in a Danish follow-up study of 113 individuals (82 males and 31 females; mean age 40.3 years) with a diagnosis of childhood autism, only one was convicted of a criminal offence (0.9%), compared with 18.9% in the control group [22]. It is important to highlight that autistic individuals may be more likely to have contact with the CJS as victims rather than perpetrators of crime [23]. For example, a literature review by Sevlever [24] and colleagues (2013) identified that difficulties recognising the emotions or intentions of others can increase vulnerability in becoming victims of sexual abuse or assault crimes.

For the small subgroup of autistic individuals who do engage in potential criminal offences, it is important to consider how specific characteristics of their autistic profile may have contributed to vulnerabilities in their pathway into offending behaviour. It is imperative to consider this on a case-by-case basis, particularly when a set of environmental circumstances outweighs an individual’s ability to cope [25]. Literature reviews and some small-scale studies have suggested that characteristics associated with autism can be linked to potential criminal offences. For example, social naivety, intense preoccupations and a narrow range of interests, theory of mind (or mentalising) differences (understanding other’s thoughts and beliefs), sensory sensitivities, emotional regulation difficulties, poor impulse control, a tendency to focus on small details and difficulty seeing ‘the bigger picture’ and the co-occurrence of other psychiatric conditions [20, 26–30]. Autistic individuals may have more difficulty making decisions by weighing up the subjective value of two different options, particularly if there is not a clear right or wrong answer [31]. This may have direct implications as to how autistic individuals navigate challenging social situations. Associated risk factors such as trauma, childhood adverse experiences and bullying are also relevant [30]. Some research has identified that the link between circumscribed interests and potential criminal offences for autistic individuals who experience them is less clear. For example, in a small UK-based case study of 21 autistic offenders (18 men and three women) [32], Woodbury-Smith and colleagues (2010) found that for 15 of the autistic offenders, the content and intensity of their interests were not be linked with their offence.

A small number of studies have indicated that characteristics associated with autism may increase the risk of engaging in sexual offences for a small subset of young people [33–36] and adults [37]. Limited evidence prohibits any concrete conclusions being drawn. However, possible increased rates of sexual offending compared with non-autistic individuals have been linked to certain features of autism, such as differences in sexual development [38–40] and/or sexual frustration due to difficulties in developing appropriate sexual relationships [35, 41].

It has been suggested that autistic traits (such as circumscribed interests and difficulties with consequential thinking, along with generally higher technical ability) rather than a diagnosis of autism may be associated with cybercrime, such as hacking [42–46]. Given the core and associated features of autism, there are also potential concerns that some autistic individuals may be more susceptible to radicalisation for several reasons, including cognitive rigidity, hyper-focusing, differences with abstract thinking, information processing and problem solving [47]. However, there is very little evidence available and more research is required.

In summary, the evidence on autism and engaging in potential criminal offences is inconclusive. Retrospective studies relying on pre-existing diagnoses (such as crime registries) may be fallible due to autism being missed or misdiagnosed [48]. Therefore, rates of offending perpetrated by autistic individuals may be underrepresented in the literature. Any link between autism and offending behaviour is likely to arise from an amalgam of individual, environmental and contextual factors.

### Prevalence of autism within the CJS

The prevalence of autism within the CJS and secure settings is unknown [49]. There are a number of explanations for this. For example, Chaplin and McCarthy [50] highlighted that autism is not part of the prison screening process in the UK [50] and there is a lack of suitable, autism-sensitive, assessment tools [25, 51, 52]. Furthermore, incarcerated populations are not static, and individuals move between institutions, creating challenges for assessments and tracking.

Many research studies exploring the prevalence of autism rely on screening questionnaires, such as the Autism Spectrum Quotient (AQ) [53], which has been shown to have poor sensitivity and specificity in clinical populations [54]. There are also limitations when using the AQ in forensic contexts due to the type of questions and nature of institutional settings [55]. Despite debates over the quality of study methodologies [21], some research has found comparable rates of autism in community and forensic populations [56–58]. However, findings are inconsistent, with some research indicating that autistic young people are more likely to come into contact with the CJS [59]. Although some consider autism to be under-recognised within the CJS [58, 60], others have reported higher rates of autism in youth and adult forensic populations compared with the general population, with rates ranging between − 4 and 4.4% [61, 62].

It is important to consider other possible influences on autistic prevalence rates in forensic settings. For example, some autistic individuals may be less able to deceive others [63, 64]. Additionally, some individuals may not be aware that their behaviour constitutes a criminal offence [65]. Existing research has predominantly focused on biological males, and little is known about the rates of autism in biological females or in specific ethnic minority backgrounds within forensic settings. Research indicates that autistic individuals from ethnic minority backgrounds are less likely to be diagnosed, receive financial benefits, such as Disability Living Allowance, or access appropriate services in the community [66]. This may be reflected within forensic populations, but further research is required.

### Multimorbidity

In both community and forensic populations, it is the norm rather than the exception for other neurodevelopmental and mental health conditions to co-occur with autism [67–71]. Co-occurring conditions include psychotic disorder, substance use disorders, personality disorder, anxiety, depression, obsessive–compulsive disorder, bipolar disorder, post-traumatic stress disorder, intellectual disability and attention deficit/hyperactivity disorder (ADHD) [48, 72–80].

Research also indicates that prisoners with elevated levels of autistic traits have increased vulnerability to mental health problems and are more likely to self-report self-harm compared with prisoners without ADHD, intellectual disability or autism [81]. Rates of suicidal ideation and completed suicide are higher than in non-autistic community populations [82, 83]. This may be influenced by a range of factors, such as genetic associations [84], restricted and repetitive behaviours [85], difficulties in communicating their experiences and having access to appropriate services [86, 87], the co-occurrence of low mood [88], sub-optimal self-esteem [89] and efforts to camouflage and conform to societal expectations and minimise stigma [90].

### Experiences during contact with the CJS

In January 2014, the Criminal Justice Joint Inspection published “A joint inspection of the treatment of offenders with learning disabilities within the criminal justice system” [91, 92] which included neurodevelopmental conditions such as autism. It found that the awareness and support for individuals with such conditions within the police, courts and prison services was insufficient. As a result, autistic individuals can be disadvantaged during their journey though the CJS [93]. Since this time efforts have been made to raise awareness of autism within these institutions, but more is required. The Criminal Justice Joint Inspection (CJJI) made a number of recommendations for improvement. These included, identifying a screening tool for universal use with the CJS, systematic collection of data to determine accurate prevalence rates and needs, the development of a neurodivergent awareness programme for staff, appropriate adjustments for neurodivergent individuals and improved coordination between different CJS agencies [94].

### Experiences with the police

Although this consensus focuses on autistic individuals who have committed potential criminal offences, it is important to note that some autistic individuals may have contact with the CJS as suspects, victims and/or witnesses. In a study of 35 Canadian autistic adults (aged 18–65) recruited from communities across Canada, 80% reported at least one lifetime interaction with police as either a suspect, victim, witness or in the context of a mental health crisis. Furthermore, 39% reported four to nine interactions with police and 14% reported 10 or more interactions [95]. In a large US sample of 920 autistic individuals (83.1% males and 67.2% white), Rava and colleagues (2017) [59] found that by 21 years of age, approximately 20% of autistic young people had interacted with law enforcement officers. In a prospective Canadian study, which followed a sample of 284 autistic adolescents and adults over a 12- to 18-month period in the community, approximately 16% had some form of police involvement during the study period [96].

Police officers have reported dissatisfaction in their management of interactions with autistic individuals. For example, Crane and colleagues [97] surveyed 394 police officers (ranging from constables to superintendents) from England and Wales. Approximately half (52%) of the police officers did not feel knowledgeable about autism and 29% reported feeling poorly equipped to manage effectively. Overall, only 42% reported feeling “satisfied” with how they worked with the autistic individuals. Autistic individuals also reported their dissatisfaction during police interactions.

The extant literature highlights difficulties at the police interview stage due differences in how autistic individuals remember and report their experiences during this socially and cognitively demanding context [98]. To date, most research has focused on autistic witnesses. However, in a recent study, Bagnall and colleagues (2023) interviewed autistic mock suspects about a novel virtual burglary scenario. Innocent (truth telling) autistic mock suspects reported fewer details that would support their innocence than non-autistic mock suspects. They also self-reported greater difficulty in understanding interview questions, had higher anxiety and perceived the interview as less supportive than non-autistic participants [99]. Similar findings were reported in a small Australian study of 32 university students (20 males and 12 females; 20–64 years) with diagnoses of autism or Asperger syndrome [100]. In the study, autistic and non-autistic adults were asked to listen to scenarios where the police believed erroneously that they had been involved in crime. Each scenario included critical information that could extricate them if the participant recognised the significance of this information and informed the police. Compared with non-autistic adults, autistic adults performed markedly worse on perspective-taking measures and the extrication task. These findings indicate that if the police erroneously suspect criminal involvement, autistic adults may have more difficulty in allaying police suspicions and extricating themselves from the focus of investigations than non-autistic peers.

The Police and Criminal Evidence Act 1984, Code C Revised [101] details that young people and vulnerable adults should be provided with an appropriate adult to safeguard their rights, entitlements and welfare. Although this legislation exists, it may not be applied appropriately and is dependent on the recognition of the vulnerability [102]. Whilst it is widely recognised that professional training about autism and managing autistic individuals’ distress whilst in police custody is important, concerns have been raised about availability of this training for police officers [103–107]. In England and Wales, police receive a 2-h mental health training programme, which includes only a subsection related to autism. As such, policy reform and improved training for police officers has been recommended to reduce distress and miscarriages of justice for autistic individuals [103].

Vulnerability in autistic individuals may be overlooked due to good expressive language skills and intellectual capability. These skills may mask difficulties in understanding, processing and responding to questions and demands during police interviews or interrogations [108]. Some autistic individuals may have difficulty understanding both the verbal and written caution that is prescribed upon arrest [109]. Understanding the police caution is integral to allow an individual the opportunity to protect their legal interests. A failure in understanding their rights may increase the risk of self-incrimination.

Due to difficulties managing the demands of police questioning, differences in receptive language ability, and additional sensory and emotional (such as anxiety) stressors during interviews, the behaviour of autistic individuals could be misinterpreted as deceitful, less credible, non-compliant, challenging or disrespectful [110–112], particularly if they have been wrongly accused or are unaware as to why they are being questioned. For other autistic individuals, misinterpretation may be due to qualitative differences in verbal and non-verbal communication, processing and/or memory difficulties, reduced cognitive flexibility, tangential thinking, Theory of Mind (mentalising) differences, repetitive movements, unusual preoccupations and perseverative interests [63].

Research indicates that individuals with ADHD (a neurodevelopmental condition with genetic, biological and behavioural features that partially overlap with autism) show more vulnerabilities during police questioning [113]. Although they are no more susceptible to interrogative suggestibility, research suggests that they are more likely to give “don’t know” responses than individuals without ADHD [114, 115]. ADHD has also been associated with higher rates of false confessions due to weakened resilience under police pressure and unhelpful attempts to end that pressure [116, 117]. By contrast there is relatively little research into suggestibility, compliance and false confessions in autistic individuals (e.g. [108, 118, 119]). The extant evidence to date suggests that autistic adults are no more suggestible than non-autistic adults (i.e., they are just as—but no more—likely to incorporate leading suggestions into their recollection of events), but they may be more compliant to (knowingly) ‘go along’ with interviewer requests and suggestions. For example, in a small UK study of 26 autistic adults (19 males and seven females; mean age 26.5 years) Chandler and colleagues [120] found that autistic individuals were more acquiescent to interviewer requests to give more of their time for free than non-autistic adults. They also scored significantly higher on the self-reported Gudjonsson Compliance scale, indicating that autistic individuals may be more likely to try and ‘please’ others by saying what they think the other individual wants to hear, irrespective of whether it is a true or accurate account of reality [120]. Similarly to those with ADHD, autistic individuals may also wish to remove themselves from the challenging situation.

Over the past 10 years, an accumulating body of experimental research indicates that standard police interviewing techniques can be ineffective in supporting autistic individuals to recall their testimony [121–123]. However, the introduction of Registered Intermediaries offers a platform for appropriate adaptations to be made during investigative interviews with autistic victims and witnesses. For instance, a Registered Intermediary can conduct a communication assessment, provide bespoke recommendations for questioning and facilitate communication during the interview process (see [124]). Currently, the Registered Intermediary Scheme in England and Wales (as outlined in the Youth Justice and Criminal Evidence Act, 1999) [125] does not apply to police suspects. Rather, the accused is specifically excluded from this provision. If autistic individuals lack appropriate support and have negative experiences during initial contact with the CJS, this may have immediate and long-term consequences for future engagement.

### Experiences of court

Following police contact, autistic suspects may move further through the CJS. They may face high levels of distress in the context of an interrogative interview or courtroom proceedings [108]. From the authors’ current experiences, the sensory aspects of the courtroom (such as lighting and noise) can cause significant sensory overload and distress for autistic individuals which may lead to negative experiences and/or difficulties with engagement. It is possible that these reactions to sensory overload are misinterpreted by jurors, the judge or prosecutor as indications of guilt or deliberate acts of antisocial behaviour (see [111, 126–130]).

Judges and juries often lack appropriate lived experience, knowledge and awareness of how autistic characteristics impact on offending behaviour, including criminal intent, behavioural control and false perceptions about potential for violence and aggression [130–132]. Legal professionals tasked with supporting their clients may also lack appropriate knowledge [133]. As a result, concerns have been raised as to whether the needs of autistic individuals are being met during criminal trials [130, 134–137] and whether juries may form inaccurate views of defendants [138, 139].

Currently, the special measures legislation in England and Wales applies solely to victims or witnesses, not suspects or defendants. These include a range of measures to support victims or witnesses, such as giving evidence from behind a screen, via video link or in private, removal of wigs and gowns by judges and barristers, intermediaries and aids to communication. Nevertheless, the courts have the power to appoint an Intermediary (referred to as ‘Court Appointed Intermediary’) if this is deemed necessary for a defendant. In this instance, an individual with suspected social communication difficulties would benefit from a comprehensive assessment prior to trial, and special measures applied where appropriate. Recommendations for interviewing vulnerable defendants and general principles for planning to question vulnerable individuals and those with social and communication needs are outlined in the Advocates Gateway Toolkits (www.theadvocatesgateway.org). Broadening legislation to support the needs of autistic suspects and defendants would improve and facilitate the process. In the meantime, advocates can seek reasonable adaptations in the court’s inherent discretion as an issue of procedural fairness. Some research suggests that a more holistic ‘trauma-informed’ approach to courts would be preferable to measures for specific categories of stakeholder [140, 141]. A trauma-informed approach acknowledges the rates and impact of trauma, seeks to understand trauma and its influence upon individuals, how it is experienced and responded to and attempts to create a ‘safe’ environment, minimising the risk of re-traumatisation. A trauma-informed approach recognises that many mental health ‘symptoms’ in survivors are actually psychological, biological and behavioural response to repeated or chronic trauma and represent intuitive coping efforts to manage traumatic distress [142]. A trauma-informed approach would involve treating everyone with dignity and respect, providing a non-judgmental approach, being mindful of how language is used and positions of power and control and making environmental adaptations. For autistic people, there is a need to consider the causes of trauma, for example the impact of sensory differences and underlying causes in the environment and a need to acknowledge and address the overlap between trauma and neurodivergence.

With regard to competency, research indicates that compared to non-autistic individuals, autistic individuals have a poorer understanding of the courtroom process which underscores the importance of appropriate support and the implementation of special measures when required (for further information see: [109, 143]. Judges consider on a case-by-case basis, whether a diagnosis of autism may negate the essential criminal elements which are required when establishing a defendant’s criminal liability or criminal responsibility. When determining criminal responsibility, two components are required, namely, (1) That the person committed the act (“actus reus”, Latin for ‘guilty act’), (2) That they had criminal intent, or intent to cause harm (“mens rea”, Latin for ‘guilty mind’). For some autistic defendants, responsibility and culpability for criminal conduct may be diminished. Berryessa [130] recommends that judges consider whether an autism diagnosis affects and negates the ability of a defendant to formulate the appropriate state of mind to commit certain criminal acts (“mens rea”). This is particularly important in cases involving specific intent crimes [130]. Specific intent crimes are crimes in which the role of the prosecution is to prove that the defendant had the desire to commit a specific crime to in order to achieve a certain outcome. The core features of autism might alter a defendant’s specific intent by undermining their capacity to form the requisite intent to harm with reference to criminal responsibility and the ability to control or project the full consequences of their actions [130, 144, 145]. Many autistic individuals are able to engage in thoughtful deliberation prior to acting. However, when some autistic individuals are in a context which is stressful, confusing or overwhelming their behavioural response may be perceived by others as aggressive ([27] as cited in [144]). These differences or difficulties in perspective taking underscore the importance of considering a defendant’s autism diagnosis when determining criminal responsibility [26, 146]—at least on a case-by-case basis.

There is a lack of research into autistic individuals being accused of complicity crimes (not acting as a principal offender but playing a role in a plan or as an accessory). It may be important to consider how an autistic individual perceives and/or understands the essential plan and their intent in the furtherance of someone else’s crime. Recent cases in England and Wales have highlighted a reluctance by courts to recognise autism as relevant to state of mind.^1^ In the absence of clear research, there is a risk of an assumption of complicity. In practice, the onus is on the defendant’s legal team to instruct experts to assess the individual and determine their understanding of the offence.

### Experiences of secure units

Conviction may result in incarceration in either prison or a secure hospital. Some research has indicated that in these settings, autistic individuals are more vulnerable to social isolation, bullying, exploitation, sexual victimisation and difficulties developing and maintaining relationships [147–149]. Communication differences can make it difficult for autistic individuals to convey their experiences to their support systems. This may impact on their support systems’ ability to meet their needs [150–152]. There is a bidirectional responsibility for both to be aware of communication differences in order to minimise misinterpretations and misunderstandings, and to maximise support. This may be more challenging for autistic individuals with a co-occurring intellectual disability, additional adaptions and support may be required to facilitate effective communication. It is important to consider that secure environments themselves, including the structure and routines provided in prisons or secure hospitals, can be supportive for some autistic individuals [152–154], but challenging for others [151, 152].

### Environmental considerations

Adapting to new environments, such as police stations, courtrooms and secure units can be particularly distressing for autistic individuals. Autistic individuals of all ages face several challenges when entering secure environments, including interacting with other prisoners, adapting to prison routines, and managing relational aggression [155]. Coupled with qualitative differences in social communication and difficulties in communicating their internal experiences and needs, autistic individuals may experience greater distress than their non-autistic peers. Minor adaptations (in accordance with secure institutions security protocols) can be made to accommodate some of these difficulties [156]. For example, for autistic individuals with hypersensitivity to noise, providing noise cancelling headphones or access to low-stimulating retreat areas may be beneficial for their overall wellbeing.

It could be argued that the prison environment is challenging and difficult for every prisoner, irrespective of diagnosis. However, for a subgroup of autistic individuals, secure environments can be particularly distressing. To date, the National Autistic Society (NAS) has accredited five prisons that have made adaptations to their environment and approach: HMYOI Feltham, HMP Parc; HMP Whatton, HMP Wakefield and HMP Peterborough. Adaptations were required to cover all aspects of prison life, including education, mental health, primary care and the prison itself. Where physical adaptations are not operationally viable or safe, autistic individuals may benefit from additional information, such as details about specific routines and behavioural expectations and safe methods of soothing, such as providing sensory resources or facilities. For many autistic individuals, ensuring that the environment and routine remains as predictable as possible may assist in minimising situational stress.

As highlighted by Newman, Cashin and Graham [151], there is increasing recognition that autistic adults in prison are more vulnerable to bullying, social isolation, sexual victimisation; exploitation or confrontations with other prisoners and to experiencing ‘meltdowns’ and ‘shutdowns’ [93, 147, 148, 157–159]. They are also more likely to be socially isolated compared with non-autistic prisoners [147]. This is unsurprising given the wealth of literature which has found that autistic individuals are more vulnerable to manipulation and bullying in the general population. Van Roekel and colleagues [149] suggest that autistic adolescents may be at a greater risk for being victimised due to difficulties in developing and maintaining social interactions and relationships; difficulties in understanding the behaviour and intentions of others [160] as well as communication differences and difficulties and stereotyped behaviour/interests that may not adhere to peer norms [161]. In turn, safety issues have raised controversies about whether autistic individuals should be extradited to face trial in other jurisdictions [162, 163].

Some autistic individuals may be allocated to special population units (e.g. vulnerable prisoner units) in an attempt to provide protection from bullying and victimisation from other prisoners [164]. For other autistic individuals, segregation or separation units may come to act as forms of behaviour management [151, 164]. However, autistic individuals may find it particularly difficult to communicate with staff about negative experiences of bullying, resulting in them not receiving access to necessary support [159]. A small Norwegian qualitative study (eight males and one female) carried out by Helverschou and colleagues [154] found that autistic individuals in forensic services did not feel confident with non-autistic prisoners, and also reported feeling different from them. One autistic individual described a preference to spend his time alone due to being teased and receiving negative comments from other prisoners.

Due to the heterogeneity of autism, and the fact that some autistic individuals may find the routine and structure of secure units quite regulating, it is not possible to provide recommendations for specific environmental adaptations. However, it is important that adaptations *are* considered, monitored and reviewed regularly. It is recognised that there may be limitations due to service resources, but support could be considered on a case-by-case basis, as needs differ for each autistic individual. Lewis and colleagues provide a clear framework for prisons to systematically work towards addressing needs for autistic individuals [159]. These standards are also in line with the NAS’s accreditation process.

### Psychosocial support and risk interventions

Non-pharmacological support is typically delivered across disciplines such as psychology, occupational therapy, speech and language therapy, education, nursing, social work and psychiatry. Characteristics commonly associated with autism (such as cognitive inflexibility, difficulties working in groups and differences in empathy) can present challenges to effective engagement and positive outcomes for non-pharmacological support and risk reduction programmes [165–167]. From the authors’ knowledge and experiences, access to appropriate psychosocial support varies greatly between services. ‘Alexithymia’ refers to difficulties experiencing, identifying and expressing emotions [156, 168, 169]. Research indicates that 40 to 65% of autistic individuals are alexithymic (e.g. [168, 170]), which is likely to contribute to and/or exacerbate core socio-emotional difficulties in autism. Alexithymia may impact on the accessibility and effectiveness of standardised programmes (for example, when individuals are encouraged to identify, address and self-regulate unhelpful or intense emotions related to their offending).

There are no pharmacological interventions for the core features of autism. In cases of extreme aggression against the self or others, the National Institute for Health and Care Excellence (NICE) guidelines recommend anti-psychotic medication [171, 172]. However, it should be noted that the purpose of such medications is to manage behavioural difficulties rather than treating core features of autism. Medication may be prescribed for co-occurring conditions such as anxiety, depression, psychosis and ADHD, and indeed this may be central to risk reduction and rehabilitation in some autistic individuals with an offending history [48, 68].

To the authors’ knowledge, there have been no empirical studies investigating the rates of reoffending or extensions to the length of stay for autistic individuals in secure settings. This gap in our knowledge places significant limitations on our ability to define and determine the effectiveness of targeted support and current care strategies for this potentially vulnerable population.

### Purpose of this consensus

Understanding and meeting the needs of autistic individuals within the CJS is essential for their care, public protection and for professionals working in forensic clinical practice. In order to develop understanding, it is important to examine and synthesise what is currently known, and to generate a professional consensus with input from lived experience on good practice when working with autistic individuals within the CJS. Given the variability of individual needs and service provisions, it is beyond the scope of this paper to provide specific recommendations. However, this paper provides general recommendations across different levels of the UK CJS.

## Methods

Twenty-four clinicians and researchers recognised as having extensive experience in the fields of autism and the CJS were invited to contribute to this consensus paper. Two potential authors did not respond and two did not have capacity to contribute due to clinical and academic demands. A further author was invited, and subsequently agreed to contribute to the paper as an author following a recommendation from a reviewer. Therefore, twenty-one professionals across a range of disciplines consented to contribute to this paper. Authors include psychiatrists, psychologists, speech and language therapists, occupational therapists, legal professionals and academics. Author experiences cover legal proceedings, academic research, diagnostic and risk assessments, delivering individual and group support and care planning for autistic individuals within the CJS. The inclusion of a multidisciplinary group ensured that the three key areas could be competently and reliably addressed. The key areas were agreed by authors EW, JH, LD and CA prior to inviting contributing authors. This followed the same process utilised by similar consensus papers [173, 174].

The three key areas included:

1. Identification and assessment
2. Support
3. Care management and multiagency liaison

Drawing on clinical experience and academic research, each author contributed towards each section which were then collated by authors EW and JH. Authors came to an agreement by consensus by approving the content of the final version of this paper. Two autistic individuals convicted of offences (SW and MK—initials included with consent) were approached to provide input based on their own experiences of the CJS. They provided written consent for their contributions to be integrated into the paper. Due to the COVID-19 governmental restrictions, at the time of writing, all communication and final consensus was agreed via email. No funding was requested or provided in preparation of this paper and neither the authors nor service users were compensated for their time in any way. This study was not pre-registered and no primary data was collected or analysed for this paper.

## Results

The authors successfully came to a consensus on guidance when identifying, managing and supporting autistic individuals within the CJS, and service user input was incorporated. Whilst the content of the consensus focuses on the UK’s CJS, the authors believe that this review and recommendations may be transferable for use in other international jurisdictions.

### Identification and assessment

#### Identifying autism

Some individuals may have an existing diagnosis of autism before they come into contact with the CJS. Autism is a heterogeneous condition and there are significant differences in the functioning and adaptive skills [175, 176]. Therefore, an assessment of their current strengths and differences may be helpful to inform an individualised support plan. It may also be beneficial to review an individual’s support needs before every transition during the CJS pathway, such as prior to police questioning, court, sentencing and incarceration.

For other individuals, autism may have been missed or misdiagnosed as another neurodevelopmental or mental health condition. This may include intellectual disability, ADHD, psychotic disorder, bipolar disorder, major depression, anxiety disorder, obsessive–compulsive disorder (OCD) and personality disorder (particularly, schizoid, narcissistic, borderline and antisocial personality disorders) [5, 177–182].

Misdiagnosis may occur due to diagnostic overshadowing, in which the primary difficulties of one condition ‘overshadow’ recognition of the features of autism. For example, ritualised and/or compulsive behaviours may have been wrongly identified as OCD, and difficulties maintaining social relationships may have been incorrectly attributed to personality disorder. Given the well-established personality disorder pathways within the prison system, there is a risk that differences may be viewed through a personality disorder ‘lens’, particularly due to the diagnostic overlaps between autistic traits and personality disorder traits [5, 183, 184]. Autistic individuals may also score higher on Factor 1, Facet 2 (affective) components of the Psychopathy Checklist: Revised (PCL-R) [185], namely apparent lack of remorse or guilt, shallow affect and callous/lack of empathy [186]. It is important for professionals to be aware of qualitative differences, overlaps and complexities, and to consider differential diagnoses within a multidisciplinary team.

Missed diagnosis can also occur when autistic individuals have developed strategies to ‘mask’ or ‘camouflage’ their social communication impairments, which is more common in autistic adults and females [187]. The structure and routine of prisons or secure hospitals may also mask difficulties associated with insistence on sameness. Camouflaging can be traced back to both learned and automatic strategies, and it is important to be aware of the habitual and unintentional dimension involved in many aspects of masking [188], particularly for females [189]. CJS professionals need to be aware that autism may present differently in biological males and females. For example, biological females may appear more verbally or socially able, have socially appropriate circumscribed interests and engage in less disruptive externalising behaviour [190, 191]. Although precise prevalence rates are unknown, these differences may partially explain the higher number of biological males referred for autism assessments in the community compared with biological females [192] and why there are subsequently more male individuals in prison diagnosed with autism than females. It has also been argued that diagnostic criteria and assessment tools for autism are based on a male phenotypical presentation [187, 193, 194], which can be associated with more pronounced autistic characteristics and/or co-occurring intellectual disability. This may lead to autism being overlooked in individuals with higher intellectual functioning.

#### Autism training for staff

Developing knowledge and understanding is dependent on access to appropriate training for professionals working within the CJS; however, opportunities for this are limited [104, 130, 139, 195, 196]. Training for all CJS professionals involved in the care and management of autistic individuals is essential and given the frequency of co-occurrence, this training could include broader training for other neurodevelopmental conditions. As recommended by the Bamford Review [195], the authors agree that autism training could adopt a two-tier approach.

In the first instance, autism awareness training would be beneficial for all professionals working with adults and young people within the CJS. The intention is to increase understanding of the condition and its heterogeneity, and support those working directly with autistic individuals to provide appropriate management and care. The Oliver McGowan Mandatory Training on Learning Disability and Autism package is available to all NHS staff. However, it is yet to be evaluated within forensic institutions. Although such training maybe helpful as an introduction to building autism awareness, it may lack the specificity required within secure settings. For example, an autistic individual who refuses to leave their room to eat in the communal dining area may be perceived as being oppositional, defiant or instrumental (doing so to achieve an ulterior outcome). They may in fact be struggling with the sensory experiences of the room or food, they may be fearful of uncertainty or the demands of social interactions and/or bullying. It is important for professionals to remain mindful of behaviour resulting from social and communication difficulties or other commonly co-occurring difficulties associated with autism (e.g. sensory differences), and disentangle this from behaviour that indicates risk to others [197, 198]. It would be helpful to evaluate the effectiveness of existing training programmes within forensic settings and/or a specific mandatory training could be developed in collaboration with the autistic community. In either case, adequate investment would be required.

The second tier includes specialist training for professionals to assess and treat autistic individuals accurately within forensic services. The SPELL framework [199], which develops understanding and strategies to support autistic individuals, has been tried with some success within secure hospitals [200] and is reported to have increased staff understanding of the issues involved in the management of autistic individuals with high levels of need. However, as this is not mandatory, it is dependent on those who are motivated to develop their understanding of autism. Specialist training can be provided by trainers with a high level of skill and knowledge in the profile of autism and the specific context in which the training is being delivered. Autistic individuals can be involved in the design and delivery of any training when appropriate, given practical and operational risk factors. In 2019, the Department of Health and Social Care [201] recommended autism training for National Health Service staff. Due to COVID-19, many training programmes have been adapted for online delivery.

#### Screening for autism

Following informed consent, a screening assessment for autism could be provided if any of the features indicated in Table 1 are identified. Screening can occur at any stage of the CJS pathway, if appropriately trained clinicians are available. All those entering the CJS may benefit from an initial screening assessment during their induction period to review their current mental health and the presence of any neurodevelopmental conditions, including autism. The presence of social communication differences identified during this initial screen could prompt further investigation to ensure individuals’ needs are met (‘secondary screening’).

**Table 1:** Indicators of the need for assessment

It is good practice for professionals working with autistic individuals to be aware of the core features of autism, including more subtle manifestations of symptoms in individuals with average or above average intellectual functioning
The presence or observation of any of the following could prompt further investigation, including a referral for a diagnostic assessment; adapted from the DSM-5 TR diagnostic criteria and ICD-11:
• Difficulties with social interaction with staff or peers, including initiation and engagement in to-and-fro conversations, reduced sharing of interests or emotions, and difficulty responding appropriately, including selective/situational mutism
• Reduced, unusual or poorly integrated non-verbal communication, such as eye contact, facial expression or understanding/use of gestures • Difficulties developing and maintaining relationships with staff and peers, including adjusting behaviour for varying social contexts
• Insistence on sameness and inflexible adherence to routines, such as extreme reactions or distress to minor changes in their routine or environment and cognitive rigidity
• Unusually intense preoccupations with subjects or objects and/or circumscribed interests that are excessive and may impact on communication or functioning
• Stereotyped or repetitive motor movements, use of objects or speech, such as repeating the words or phrases of others (echolalia) or using stereotyped or idiosyncratic language
• Unusual sensory interests or sensory sensitivities, such as hyper and hypo sensitivity and/or sensory avoidance/seeking
• The presence of other neurodevelopmental conditions, such as ADHD and intellectual disability
• A family history of neurodevelopmental and/or genetic conditions with known associations with autism
• An existing diagnosis of OCD or the presence of obsessive/compulsive traits
• Existing diagnoses of personality disorder, particularly schizoid personality disorder, obsessive–compulsive personality disorder and/or personality disorder – not otherwise specified
• Highly detailed offence accounts combined with indicators of poor episodic memory and/or sequencing difficulties

#### Initial screening for young people

A wide range of tools are used to identify young people’s needs, within Secure Training Centres and Youth Offender Institutions (YOIs). The National Health Service (NHS) England recommends YOIs use the Comprehensive Health Assessment Tool (CHAT) [202]. The CHAT is a validated semi-structured assessment for use with young people within YOIs. It includes exploration of physical health, substance misuse, mental health and neurodivergence, including autism, ADHD, intellectual disability and traumatic brain injury. However, this assessment is not compulsory and may be refused by the young person. The effectiveness of initial assessments is dependent on the skills of the assessor and their ability to interpret responses and identify areas requiring further investigation.

#### Secondary screening for young people

Social communication difficulties identified by the CHAT may indicate the need for further assessment. For young people with available informants such as parents or carers, the Social Communication Questionnaire (SCQ) Current or Lifetime Forms [203, 204] could be used. The SCQ is a parent report questionnaire containing 40 items with good diagnostic validity [205] and is used for young people over the age of four. To the authors’ knowledge, there are no validated self-report autism screeners for young people.

Due to the high rates of co-occurring conditions, it may be useful to administer additional screening tools to inform thinking around autism and co-occurring/differential diagnoses. This may involve screening for the most frequently observed co-occurring conditions such as anxiety, depression and ADHD [69, 206]. An eight-item questionnaire on quality of life (subjective wellbeing), the Personal Wellbeing Index (PWI) developed by Cummins and colleagues (2005) [207] at Deakin University Australia, has shown to be useful for measuring the quality of life with autistic young people and adults.

#### Initial screening for adults

To the authors’ knowledge, there are no government-mandated assessments for adults on admission to prisons or secure hospitals. Within secure services, adults may access psychiatric reviews and support through self-referral or staff referral. The CJJI’s [94] review of the evidence has recommended utilising a screening tool for universal use within the CJS and that outcome data should be systematically collected to inform service planning at all levels of the CJS.

#### Secondary screening for adults

Many existing screening tools take the form of self-report questionnaires, which can be problematic, particularly for individuals with social communication differences. The Autism Spectrum Quotient, 10 items (AQ-10) [208] is a screening tool for adults and is currently recommended in the NICE guidelines for use in the general population [209]. This is a shortened version of the AQ-50 [53] and requires the individual to rate the extent to which they agree with ten statements on a 4-point Likert scale. This includes metaphorical statements such as “I find it easy to ‘read between the lines’ when someone is talking to me”, which some autistic individuals may find confusing. In order to make this judgment, the individual would require a reasonable degree of personal insight along with an awareness of situations in which they have ‘missed’ implied meanings. Many autistic individuals may find it difficult to identify nuances in social situations, which has inevitable implications for self-report screening questionnaires. Supplementary information, such as observations from informants or previous reports, could be used to inform the decisions about the need for further assessment. If self-awareness is limited and/or the individual is unable to provide an accurate appraisal of their functioning and presentation, self-reports may be used alongside observations from CJS professionals who know them well. The AQ-10 and AQ-50 have been identified to have limited effectiveness, particularly with clinical and forensic populations [54, 55, 210]. If the AQ-50 is used, Murphy [55] recommends that the measure is adapted into a semi-structured interview to accommodate for the poor literacy skills frequently identified in forensic populations.

The Social Responsiveness Scale, Second Edition (SRS-2) informant and self-report forms [211] may be a helpful screening tool for adults. The SRS-2 is a 65-item validated measure for adults aged 19 and above and covers four key areas including Social Awareness, Social Cognition, Social Communication and Social Motivation that can be summed into the domain of Social Communication and Interaction. Additional scores are summed to calculate the domain of Restricted Interests and Repetitive Behaviour. Together both domains can be combined to calculate an overall Social Responsiveness Scale score. The informant version can be completed with a family member, partner, friend and/or member of staff who knows the individual well.

The Communication Checklist – Adult (for ages 17–19) [212] and Communication Checklist – Self Report (CC-SR) [213] (for ages 10–80) may also be useful during the screening process. These tools are used routinely by Speech and Language Therapists to screen for communication impairment and subtle communicative differences which may be seen in the broader autism phenotype.

Additionally, the Ritvo Autism and Asperger Diagnostic Scale-14 (RAADS-14) [214] is a 14-item self-evaluation questionnaire based on the 80-item Ritvo Autism and Asperger Diagnostic Scale-Revised [215]. This measure was designed to differentiate between autism and other psychiatric conditions. This screening tool has been identified to have good sensitivity and specificity within psychiatric populations [214] and has been recommended as a useful starting point within forensic populations to determine whether a more comprehensive assessment is required [216]. However, the potential limitations to the RAADS-R should be acknowledged [217]. See Appendix 1: Table 4 for table of relevant secondary screening tools.

#### Diagnostic autism assessments for young people and adults

Accurate identification of autism or the confirmation of an existing diagnosis is fundamentally dependent on the resources available within each stage of the CJS. Comprehensive autism assessments require access to records, specialist staff trained in relevant diagnostic tools, and information from caregivers or informants. It also relies on the individual engaging in the assessment process. The decision to go ahead with an assessment may depend on an individual’s understanding and perception of autism as a condition, social and cultural influences, (dis) trust of the CJS and/or health professionals and perceived advantages/disadvantages of the assessment process and the implications of a potential diagnosis.

**Fig. 1:**
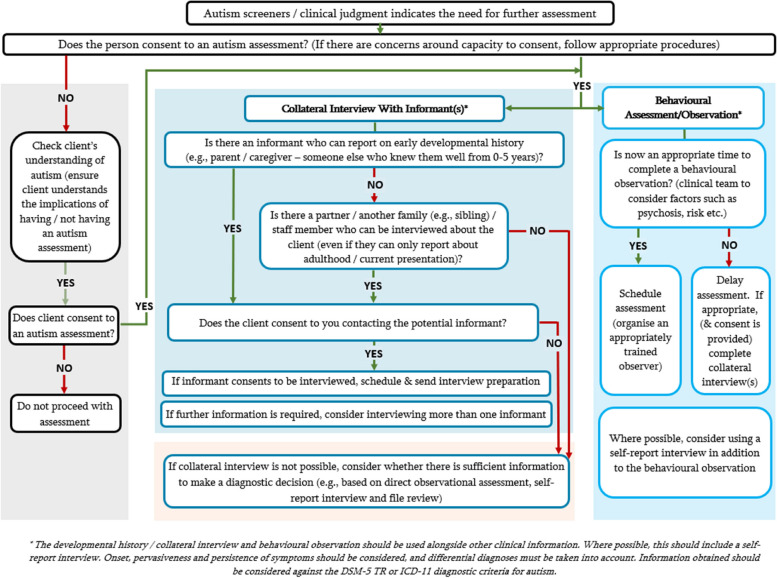
Diagnostic flowchart

Wherever possible, the assessment of autism should include a detailed autism-specific developmental history, which is usually completed by a family member. This is critical in determining the onset, pervasiveness and persistence of characteristics, and assists clinicians in their consideration of differential diagnoses. Comprehensive autism-specific developmental histories can be difficult to obtain, particularly in situations involving complex dynamics between autistic individuals and their family members [218–220]. For example, family involvement may be difficult if family members have been the victims of the offending. Recalling specific information about early development from a long time ago may also be difficult for family members. An autism diagnostic flowchart has been developed for this paper and is provided in Appendix 2: Figure 1, detailing alternate strategies for obtaining information from informants.

The authors agree with guidelines laid out by the NICE [171, 172] that every diagnostic assessment include:

- A comprehensive autism-specific developmental history in accordance with the International Statistical Classification of Diseases and Related Health Problems 10th Revision and 11th Revision [4, 221] or Diagnostic and Statistical Manual of Mental Disorders 5th Edition Text Revision [5]. This should also include a family medical history and the individual’s experiences of home life, education and social care.
- A behavioural observation of current strengths and difficulties associated with autism.
- A physical examination and/or physical health review, for frequently co-occurring physical health conditions, if appropriate.
- A psychiatric review investigating the presence of co-occurring conditions and conducting differential diagnosis.

It may be necessary to complete an autism-specific observation of the individual within a social context (for example, during a group activity, a work role or during free time). In addition, an age-appropriate cognitive assessment may be useful to determine a profile of current cognitive strengths and differences. It is common that autistic individuals present with a ‘spiky’ or ‘uneven’ cognitive profile. This can lead to incorrect assumptions about the capabilities of autistic individuals. For example, autistic individuals with fewer verbal abilities may be assumed to be lacking in other skills or potential, which may not necessarily be the case [222]. Alternatively, autistic individuals may have a high level of expressive language which masks relatively poor understanding.

Additionally, someone’s intelligence quotient (IQ) is not always commensurate with adaptive functioning (such as daily living skills, communication and social skills). Autistic individuals without intellectual disability can experience adaptive functioning skills far below what would be expected given their intellectual potential [223, 224]. Therefore, an additional assessment of adaptive functioning for autistic individuals without intellectual disability would provide essential insights into needs and possible support strategies [225]. The Adaptive Behaviour Assessment System, Third Edition (ABAS-3) [226] enquires about adaptive skills related to self-care, responsiveness to others and the ability to meet environmental demands.

Given our current understanding of sensory differences, reflected in the DSM-5-TR and ICD-11, some standardised assessments which were developed prior to the introduction of these diagnostic manuals (such as the ADI-R) may not accurately capture the full range of sensory differences. Therefore, we suggest that an additional sensory measure is used to supplement the assessment process, such as the Sensory Processing Measure, Second Edition [227]. This tool explores a range of different sensory systems, including visual, auditory, tactile, olfactory and gustatory, proprioceptive and vestibular. We also suggest enquiring more about interoceptive sensory differences.

As previously stated, both neurodevelopmental and mental health conditions frequently co-occur with autism. Therefore, consideration of co-occurring mental health conditions should form an essential component of the autism assessment and subsequent care planning [228, 229].

#### Autism-specific developmental history

Whilst autism-specific developmental histories can be completed without the use of a validated semi-structured interview, it is good practice for established and validated measures to be used to ensure accuracy and consistency. There are seven recommended diagnostic interview tools that are suitable for assessments with both young people and/or adults: the Autism Diagnostic Interview – Revised (ADI-R) [230, 231], the Developmental, Dimensional and Diagnostic Interview (3di) [232], the Diagnostic Interview for Social and Communication Disorders (DISCO) [233], the Diagnostic Autism Spectrum Interview (DASI) [100], The Asperger Syndrome and high-functioning autism diagnostic Interview (ASDI) [234], The Autism Clinical Interview for Adults (ACIA) [235] and the Royal College of Psychiatrists Interview Guide for the Diagnostic Assessment of Able Adults with Autism Spectrum Disorder – Revised Edition [236]. The choice of diagnostic tool may be dependent on available resources. Further information about tools to record developmental histories are reported in Appendix 3: Table 5.

It is important that the autism assessment process involves at least one professional who is qualified to assess differential diagnoses and/or co-occurring conditions. Before making a diagnostic decision, it is essential for clinicians to consider whether the quantitative and qualitative information obtained through diagnostic tools could be better explained by another condition (for example, trauma, anxiety, psychosis, personality disorder). Clinicians completing autism diagnostic assessments should have experience of autism and other neurodevelopmental conditions, as well as experience of working in forensic settings. Given the complexity of forensic populations, specialist training in standardised tools is recommended. Some diagnostic tools include algorithms which indicate whether the individual scores above or below diagnostic thresholds. Whilst diagnostic thresholds can be used as guidance, quantitative scores should be interpreted with caution. Qualitative information obtained from multiple sources along with clinical judgment from a multidisciplinary team is essential when forming diagnostic conclusions in this complex clinical population. It is important to distinguish the overall clinical diagnosis from the outcome of one specific diagnostic tool. The overall clinical diagnosis requires consideration of multiple sources of information alongside an in-depth clinical knowledge of autism and differential and/or co-existing clinical conditions [237–239].

#### Autism behavioural observations

NICE guidelines stipulate that a behavioural assessment (including interaction and direct observation) be completed to gather objective information about the individual’s current strengths and differences. Whilst it is not a requirement that a standardised and validated tool is used, it is best clinical practice to use such a tool to ensure accuracy and reliability. Behavioural observations should be completed by professionals who have been trained to undertake such observations and have experience working with autistic individuals.

The Autism Diagnostic Observation Schedule, Second Edition [240, 241] is a standardised and validated assessment tool comprised of activities and questions that provide opportunities for the individual to demonstrate social communication strengths and difficulties. Considered to be the “gold standard” [238, 242], the administrator can choose from five modules depending on the individual’s age and expressive language level.

Assessors are required to create a nuanced social interaction within a semi-structured, standardised framework, and to assess the interaction simultaneously by making 29–41 highly specific observations (depending on the module). Due to the complexity of the administration and scoring, the ADOS-2 should only be administered by clinicians who have received formal training in the ADOS-2. To be considered ‘reliable’ in the assessment, assessors must consistently demonstrate a minimum of 80% reliability (agreement with consensus scores established by trainers). Further information can be found in the ADOS-2 manual [240, 241]. Where possible, the ADOS-2 could be filmed and/or observed by a second ADOS-2 trained clinician, and this is particularly valuable when working with complex populations. However, it is acknowledged that there may be barriers to filming within some forensic settings.

#### Physical examination

A standard physical examination typically includes a review of the individuals vital signs (blood pressure, breathing rate, pulse, temperature, height and weight), vision acuity, head, eyes, ears, nose and throat, gastrointestinal health, cardiovascular, respiratory, and musculoskeletal assessments, neurological examination (mental awareness, motor function and balance, sensory response and reflexes) and a skin and lymph node check.

In addition, NICE [172] recommend a specific focus on skin stigmata of neurofibromatosis or tuberous sclerosis using Wood’s light, signs of injury (self-harm or child maltreatment) and congenital anomalies and dysmorphic features including macrocephaly or microcephaly.

#### Psychiatric review

A psychiatric review is an opportunity to learn more about the individual’s perspective. NICE [172] recommends that an autism-specific psychiatric review include a family medical history, consideration of differential diagnosis and the systematic assessment for the presence of co-occurring neurodevelopmental or mental health conditions, medical or genetic disorders and functional problems (e.g. restricted diets, bladder or bowel conditions, sleep disturbance and/or vision or hearing impairments). Following the psychiatric review, referrals can be made for additional assessments and/or support, if required.

### Risk assessment for autistic individuals

There is a growing recognition that current standardised risk assessments, such as the Historical-Clinical-Risk Management-20, Version 2 [243] and Version 3 [244] have limited use with autistic individuals [25, 51, 245–247] given they are normed on the general population.

Inappropriate assessments may lead to professionals either over- or under-estimating an autistic individual’s level of risk (e.g. [187, 246, 248]). There are additional complexities in assessing risk for autistic individuals, including inherent social communication differences, variation in presentations and co-occurring conditions, which may affect autistic individuals differently [51, 52, 247]. Therefore, it is recommended that structured risk assessment tools such as Version 3 of the HCR-20 [244] and the Risk of Sexual Violence Protocol Version 2 (RSVP-V2) [249] should also provide an adjunct individual formulation. Given the limitations of current risk assessments for autistic individuals, we suggest that the tools applied are carefully considered and assessments are conducted by professionals with specialist training in autism.

To date, no risk assessment tools have been validated for use with autistic individuals. However, the Framework for the Assessment of Risk and Protection in Offenders on the Autistic Spectrum (FARAS) [198] can be a useful clinical tool. The author clearly indicates that the FARAS must not be used to replace standardised risk assessments, but as an adjunct to and to supplement standardised risk assessments to address the autism-related functions of areas of risk and protection. It is recommended that professionals either familiarise themselves with the tool or undertake specific FARAS training before use. It should only be used by those who have completed training in formal risk assessment. The FARAS is organised in seven sections based on features of autism that relate to risk factors and protective factors in offending behaviour and offers guidance on how each facet can be considered during the interview. The information obtained from the FARAS should be integrated into the formulation of risk and protection, and may inform support and risk management strategies.

An autism-appropriate risk assessment should include consideration of co-occurring mental health conditions, social awareness, cognitive and socio-emotional intelligence, vulnerability to exploitation or sexual victimisation, stereotyped behaviours, unusual or atypical preoccupations and self-harm and/or suicidality. An individualised review of protective factors should also be considered, such as the impact of the immediate environment, given the difficulties some individuals may have managing changes in their environment and sensory differences (see Gunasekaran, 2012) [51].

Evidence suggests that differences in social cognition account for a substantial amount of social communication difficulties for autistic individuals [250]. Social cognition is associated with the perception, processing and interpretation of social interactions. Differences in explicit and implicit social cognition may inform our understanding of an individual’s risk. The Dewey Story Test [251] requires individuals to judge eight sample social situations and determine how they would respond. It can inform our understanding of how an individual is seeing the world, including complex social relationships. This may need to be considered differently depending on whether the accused individual is alleged to be a principal or complicit offender.

When communicating risk assessment findings to autistic individuals, conclusions should be presented clearly and concisely. The individual’s presentation and social communication during the risk assessment may inform clinical thinking about the context of their autistic functioning and the most appropriate, effective way to provide feedback. For example, a detailed, matter-of-fact offence account which appears to lack social and emotional sensitivity may reflect the individual’s detailed and literal cognitive and communication style, differences in Theory of Mind (mentalising) and qualitative differences in non-verbal communication.

### Support

Due to the heterogeneity within autism, a ‘one-size-fits-all’ approach cannot be utilised. Following an autism diagnosis, a review of the individual’s strengths and differences should inform support strategies that are appropriate for their specific profile of needs.

For convicted individuals with an existing autism diagnosis, it is important that there is continuity of care as they transition from community services to forensic services. It cannot be assumed that individuals who were diagnosed with autism in the community have received adequate psychoeducation or support, particularly as many out-patient diagnostic services discharge individuals once the assessment process has been completed. Therefore, it may be helpful to consider a review of strengths and differences before tailoring management, assessment and rehabilitation approaches.

Access to appropriate support for autistic individuals relies on available resources, including appropriately trained clinical staff. This may vary between different facets of the CJS, which is not surprising given that recommendations for support typically follow the identification of impairment or difficulties (such as a conviction for an offence). In line with government guidelines, some programmes within secure services and probation settings are accredited by the Correctional Services Accreditation and Advice Panel (CSAAP) and their delivery must adhere to specific standards. Some programmes are flexible enough to allow for specific adaptations to ensure individual responsivity. However, when the autistic individual’s risk, needs and responsivity cannot be addressed through accredited programmes, individualised alternative support may be considered.

Rehabilitation is much broader than specific psychosocial support and includes all aspects of the individual’s day-to-day living. In this respect, autism awareness training may be helpful for all those involved in the individual’s care and support, including professionals making decisions about the individual’s case. This includes solicitors, prison officers, educators, clinicians, probation and The Parole Board. Based on our knowledge and experience, this level of awareness is not universal across the CJS.

Overall, evidence for the effectiveness of support for autism is inconsistent. This may contribute to the apprehension or concern with which some autistic individuals may approach recommended support. Possible exceptions include those associated with supported employment [252] and more recently those concerning mentoring or life-coaching [253].

#### Psychological support

Numerous programmes have been developed across different domains with varying degrees of effectiveness [254, 255]. Following an autism diagnosis, it is good practice to provide psychoeducation and for a formulation of the individual’s specific strengths and differences to be discussed collaboratively. Psychoeducation can also be helpful for the families of autistic individuals if a diagnosis has been received during incarceration.

Psychoeducation aims to provide the individual with insight into their own social communication, sensory and neurocognitive strengths and differences [256], and increase engagement in support. Psychoeducation is generally recommended as part of good post-diagnostic care [171, 172] but may also be helpful in increasing an individual’s insight into how their particular set of strengths and differences may impact upon both risk and resilience.

Psychological support may be provided based on an individual’s specific needs. There is a growing evidence base for the effectiveness of psychosocial support addressing the core features of autism and associated difficulties (such as activity scheduling, relaxation and anxiety management) within community-based populations [257, 258]. However, there is little research as to the effectiveness of psychosocial support within the CJS. The National Autistic Society has developed a good practice guide for professionals delivering talking therapies for autistic adults (https://www.autism.org.uk/).

#### Cognitive behavioural therapy (CBT) for co-occurring mental health conditions

Whilst adapted CBT can be effective for autistic individuals [259, 260], it is important to recognise that changing cognitions may be more difficult than it is for non-autistic individuals [166]. Hare [261] recommends that cognitive changes should not be a primary goal in CBT approaches for autism. Instead, a focus on behavioural adaptations more practically equips the individual to manage daily living.

Overall, CBT programmes adapted for prisoners with developmental differences show similar outcomes compared with CBT programmes in community populations [262–264]. However, this research included a range of neurodevelopmental differences rather than autism specifically.

#### Educational and occupational support programmes

There is substantial variability in the academic performance and attainment of autistic individuals [265]. Therefore, it is important that autistic individuals of all ages are supported to engage with appropriate academic and occupational programmes during their incarceration and following their release into the community. Research has identified that education and employment opportunities are some of the most requested needs from individuals being released from forensic institutions [266]. However, the type of employment needs to be stable and of a type and duration that is compatible with their preferred lifestyle [267]. This is of particular importance given additional challenges faced by autistic individuals within the work environment. Data published by the Office for National Statistics [268] reported that only 29% of autistic individuals aged between 16 and 64 were employed in the UK, which is far below their non-autistic peers. The number of research articles examining neurodiversity within the workplace is growing [269] and potential barriers and enablers are being identified [123, 270, 271]. However, less is known about the possible cumulative effects of being both autistic and having criminal convictions with regard to employment opportunities. A small Norwegian study found employment rates of 27% for autistic adults in contact with a forensic service between 2000 and 2010 [272] but more research is required, particularly in the UK. Occupational opportunities could be identified that best suit the needs and strengths of autistic individuals, whilst also considering risk [152]. For example, construction work is unlikely to be helpful for an individual with sensory sensitivities to sound, but if a role involves their special interest or passion, they may be more likely to engage successfully. Upon release, autistic individuals may require continued support in their chosen occupation. This support could be co-ordinated between the individual, employers and local community forensic teams, and CJS case managers.

In addition to supporting individuals to develop daily living skills, appropriate Occupational Therapists can also provide bespoke sensory assessments for those that experience sensory differences. As previously described, sensory differences can have a substantial impact on autistic individuals throughout their CJS journey. Determining how an autistic individual processes sensory experiences can lead to helpful environmental adaptations and support managing sensory stimuli.

#### Speech and language

Some autistic individuals may experience speech and language differences. Speech and language differences in childhood may persist into adolescence and adulthood [273] and can negatively impact on educational achievements [274]. One study suggests up to 60% of young offenders present with speech and language difficulties [275]. This may be a direct risk factor for engaging in potential criminal offences in biological males due to differences with social interactions and understanding [276, 277], although little is known about the impact on biological females. It is recognised that these difficulties may be poorly addressed within the CJS [278]. Speech and language differences will continue to create challenges for individuals navigating the complex language and terminology within the CJS. Two significant protective factors include family/friendships [279] and employment [280], both of which require substantial language and communication skills. Therefore, for those that require it, speech and language therapy/therapist involvement is recommended to ensure that autistic individuals can access support that is necessary to assist with rehabilitation and progression through the CJS, including supporting autistic individuals to understand any licence requirements for resettlement or their transition to the community.

#### Prison programmes

Offence-related interventions are often cognitive-based programmes delivered in a group format. It is important for professionals to be aware of the potential limitations of these for autistic individuals. Whilst prisoners with neurotypical presentations can be expected to integrate in conventional therapeutic programmes, it is likely that some aspects of the support process fall short of meeting the needs of autistic individuals. For example, group-based support requiring interaction with peers and facilitators, emotion-relate work and content relying on auditory processing may be especially challenging for autistic individuals to engage with and complete programmes. This is relevant to group work addressing sexual offending [156, 281].

Higgs and Carter [281] highlight that many programmes addressing sexual offending begin by seeking to establish group cohesion, pro-change norms and positive relationships between group participants, which requires social interaction. This necessity for social interaction means that some autistic individuals may experience greater difficulty with group processes than their non-autistic peers. Descriptions of offences provided by autistic individuals may be limited to the factual reporting of events, and insight into different interpretations of the offence and acknowledgement of inappropriate behaviour can be difficult to achieve [156, 282]. Other autistic individuals may have difficulty sequencing information in a logical and coherent manner [283] and require support from speech and language therapists to structure their narratives [284].

The interpretation of support outcomes is an important factor when considering rehabilitation progress and parole hearings. For example, in a study of autistic individuals with sexual offence convictions, Vinter [156] identified that staff may under- or over-estimate progress and risk. Staff reported that autistic individuals may appear to have made good progress by using appropriate therapeutic language, but may simply be repeating specific programme terminology without an understanding of the deeper meaning. Staff also suggested that some autistic individuals may have made genuine progress but struggle to communicate their understanding effectively.

Previous risk reduction programmes were not designed to cater for the differences of autistic individuals and may have disadvantaged them in their progression towards parole and release [285]. Individualised interventions are more likely to be effective in addressing specific areas of risk, needs and responsivity. Some considerations are presented in Table 2.

**Table 2:** Considerations for adaptation during psychosocial support

Where possible, we suggest that 1:1 support or supplementary 1:1 support is provided. Consideration could be given as to whether the individual would prefer to engage in-person or on-line. The following adaptations may be helpful when working with autistic individuals of all ages:
• Introduce and maintain a structured approach (e.g. frequency, duration and timings of sessions)
• Consider using the three ‘V’ approach to structure: visual schedules, visual instructions and visual clarity
• Ensure that the rules of the individual or group work are understood
• Use clear, concrete language and avoid abstract language
• Provide time and space for the individual to process new information
• Consider whether some autistic individuals need more sessions and more time to apply and generalise skills in between sessions
• When establishing rapport and working therapeutically, alternatives to verbal communication may be helpful. Where feasible, this might include communicating via music, art or computer games
• Behavioural, rather than cognitive work may be more effective, when applicable. However, some autistic individuals will respond well to the detailed theoretical and logical analysis, which can be critical in some interventions
• Incorporate healthy special interests/passions when appropriate, as sources of reward and wellbeing. Manage risky special interests/passions and develop the individual’s skills to do this
• Adapt materials to attend to autism learning styles such as visual learning, modelling with practice, social stories and appropriate handouts to summarise and integrate the learning
• Create an environment that attends to possible sensory sensitivities (e.g., minimising noise,
• artificial lighting, strong smells, need for movement)
• It may also be helpful to keep work areas clutter free and quiet
• Remain mindful of frequently co-occurring conditions such as anxiety and low mood, which may affect motivation and engagement during intervention
• Remain mindful that limited verbal and non-verbal communication (such as limited eye contact or facial expressions, lack of expressed victim empathy, insight into the emotional states of self and others and insight into typical social relationships) should not be misinterpreted as non-compliance, disengagement, or deception
• Pre- and post- intervention outcomes may need to be adapted to the needs and abilities of the individual. This may be different from neurotypical individuals
• Consider the individual’s ability to identify, understand and communicate their own emotional experiences. Use visual rating scales, emotional scales and feelings boards
• Support to apply and generalise learning to new contexts and situations that look different from the offence or current predicament

#### Pharmacological support

A comprehensive review of pharmacological options for autistic individuals is beyond the scope of this paper. However, we have identified some important considerations related to this topic.

It is important to state that there is no medication to support the core features of autism and pharmacology is not recommended for this purpose [286]. Some behaviour that may be perceived as aggressive towards the self and/or others can be understood as the involuntary physical and emotional reaction to stressors that overload the nervous system [287]. As such, situational and/or environmental changes ought to be the first response, prior to medication. However, in extreme cases pharmacology can be considered to manage aggressive and/or self-harming behaviour when other non-medical adaptations have not worked [171, 172, 288].

The primary role of medication for use with autistic individuals is to support them with associated difficulties and/or co-occurring conditions [289, 290]. For example, selective serotonin reuptake inhibitors can be used for anxiety, low mood and repetitive behaviours [291], psychostimulant medication for associated hyperactivity [292], and atypical antipsychotics to manage irritability [293], aggression, self-injurious behaviour and ‘meltdowns’ [294–296]. However, it should be noted that many drug trials specifically exclude autistic individuals or do not clearly identify them. Therefore, there is limited understanding as to the effectiveness of medication for autistic individuals.

It is important that a psychiatric and physical review is completed before the prescription or administration of any medication for any condition. A medication review of previous medications, indications, doses, effects and side effects should be undertaken prior to the introduction of medication. Prior to the administration of medication, the autistic individual should be informed as to the purpose and possible side effects of medication and their consent obtained. In order to facilitate this, information could be provided in an accessible format. They should also be informed as to the implications of remaining unmedicated, non-compliance and the appropriate means of ending medication. Prescription protocols should be consulted accommodating age, gender, physical health, pre-existing conditions and pregnancy.

Given the sensitivity of some autistic individuals to the effects and side effects of medication [297], a ‘start low, go slow’ approach could be adopted. In practice psychotropic medications can be introduced at 25 or 50% of their normal starting dose, and increased in small increments at intervals (e.g. weekly) following review of symptoms, effects and side effects. Regular medication reviews should be completed to enable effective monitoring and management of responses to pharmacological interventions and co-occurring conditions. Systematic parameters to evaluate the effectiveness of these interventions should be agreed with the individual. The determination of treatment effects and side effects should be informed by systematic staff observations and self-report. It is important to bear in mind that social communication difficulties and/or alexithymia may impact on an autistic individual’s ability to report on the immediate and broader effects and side effects of medication.

### Care planning and multiagency liaison

In 2000, the Department of Health assumed responsibility for healthcare provision within Her Majesty’s Prison Service, with full administrative control completed in 2006. Since this time, several improvements have been reported, including improved healthcare services in prison, increased engagement of service users ‘voice’ in service development and improved continuity of care for people as they transition from prison to the community [298]. In addition, prison and probation services are required to provide a level of social care and support *equal* to that of people living in the community [299].

Regarding the management of young and adult autistic individuals within the CJS, we recommend that a ‘shared record’ of the individual’s needs is communicated to all agencies involved throughout their journey within the CJS. Some potential challenges for professionals working with autistic individuals are identified in Table 3.

**Table 3:** Potential challenges for professionals working with autistic individuals

The following is a list of possible challenges faced by professionals working with autistic individuals. The purpose is to encourage consideration of autism related challenges rather than negative or unhelpful interpretations of behaviour, such as non-compliance or disengagement
• Qualitative differences in social communication may lead to difficulties in developing meaningful relationships, including relationships with professionals and pro-social peers. This may also have implications for future placements, such as the appropriateness or effectiveness of supported accommodation or shared rooms
• Professionals may experience compassion fatigue, particularly in the face of cognitive rigidity. This can change the way professionals work with individuals and lead to staff burnout and compromise the therapeutic relationship. Debriefing and reflective practice may reduce these experiences
• Due to physiological arousal linked to sensory or social overload, autistic individuals may become involved in confrontations or altercations with others. In contrast, autistic individuals may prefer solitude, and this should not be perceived as disengagement or non-compliance with staff or prison expectations
• The individual may have difficulty working out appropriate or effective solutions to problems, engaging in long term planning or setting unrealistic goals for the future due to a particular thinking style or may take things literally
• Communication should be explicit and non-ambiguous. Refrain from using abstract concepts. Provide opportunities for individuals to clarify whether they have understood. If not, then information should be presented through different mediums
• Learnt phrases/expressions and uneven cognitive profiles may mask social communication difficulties. It is important to check the individual’s understanding of information provided, and ensure that this is available in written (or other visual forms) if this is beneficial
• Differences in non-verbal communication may mean that professionals find it difficult to understand, identify and respond to changes in the person’s emotional states. This can impact on social and emotional interactions, and lead to misunderstandings. For example, an autistic individual may have difficultly modulating the volume of their voice, use unusually intense eye contact and stand too close to others due to difficulties understanding personal space. Some individuals may use incongruent facial expressions (for example, smiling when talking about a serious or distressing topic) or attempt to use humour when this is socially inappropriate. These behaviours might be interpreted as aggressive, deliberately intimidating and/or callous when they are in fact a result of social communication difficulties associated with autism. It may be helpful for professionals to tell autistic individuals that they do not have to make eye contact if this is easier for them
• Sensory modulation difficulties may impact on the autistic individual’s ability to engage with regimes and this should not necessarily be perceived as defiance. The environment should be audited for potential stressors around proxemics (people), noise, heat, odours and bright lights. These can be extremely distressing and provoke extreme reactions in autistic individuals. Ask the individual whether there is anything that is distressing them

Based on individual needs, custodial alternatives could be considered along with joint working with local community forensic teams. In such cases, appropriate support could be made available. This may require collaborative work between secure services and community services, including the sharing of knowledge, experience, tools and education. In addition, courts will require information about the evaluation of the effectiveness of such support.

Local multiagency strategic planning groups could be developed between autism and criminal justice services. Representatives who have received specialist training from each service (such as police and prison health care staff) could follow the individual throughout their CJS journey [300]. These individuals and groups need to be fully integrated into the care plan to ensure effectiveness.

Individuals with mental health needs receive a Care Programme Approach [301, 302] within secure units. A CPA is a package of care that covers a range of domains (such as risk and support strategies) and requires the input from a multidisciplinary team. A CPA care co-ordinator is allocated to prepare and manage the individual’s care plan and review it at least once a year [303]. Other jurisdictions within the UK may have similar processes, such as Care and Treatment Planning, in Wales. For autistic individuals with co-occurring mental health needs and receiving CPAs, we encourage these to be reviewed at least every 6 months, and/or in advance of a significant change (such as release), and/or in response to a significant incident (such as being the victim or perpetrator of violence). The absence of structured support such as CPAs could disadvantage or limit progression. In addition, autistic individuals with co-occurring mental health needs would benefit from regular psychiatric reviews to identify, monitor and manage their mental health needs.

Given that major transitions may be challenging for autistic individuals [304, 305], attention could be given to all transitions from initial contact with the CJS to integration back into the community. A critical time intervention approach [306] in which a designated individual meets with the individual prior to their release and manages their care plan in the community could be considered. This could include liaising with relevant local services and their general practitioner.

It may be beneficial to refer autistic individuals to locality Forensic Outreach Liaison Services (FOLS) within England, and other jurisdictions may have similar services (e.g. Supporting Offenders with Learning Difficulties (SOLD) in Scotland). These are multidisciplinary NHS services working with autistic individuals and/or those with intellectual disability in the CJS, including those who have been released. Where identified needs are linked to autism and/or intellectual disability, FOLS can provide direct intervention and support. The Forensic Intellectual and Neurodevelopmental Disabilities (FIND) services that form part of FOLS can provide risk assessment reports tailored to the needs of autistic individuals. FIND services offer an experienced multidisciplinary team approach (including psychiatry, psychology, nursing and social work) to complete access assessments and care pathway monitoring for those transitioning into and out of secure services. FIND services can also offer individuals and services assessment advice, training and information when transitioning out of secure care. Depending on the individual’s needs, this can be extended for a short period in their new placement.

## Discussion

Some autistic individuals may experience challenges across different levels of the CJS. The current processes and pathways for assessment, support and management of autistic individuals within the CJS are inconsistent [91, 92]. The authors provide a review of the literature and possible gaps in the current management of autistic individuals. These include an inconsistent awareness of autism across different levels of the CJS, absence of agreed diagnostic assessments and effective care and management plans, insufficient specialised training for the assessment and support of autism and inadequate management within and following release from secure services. We also provide general recommendations for the assessment and support strategies for autistic individuals.

Ideally, the authors envisage an individualised care and management plan that adopts a multimodal approach. For those with an existing autism diagnosis, this would start from their initial contact with the CJS and continue throughout their journey and beyond their transition to the community. If autism is identified whilst they are under the care of the CJS, this individualised care and management plan could begin immediately following their diagnosis.

Whilst the authors have developed this review based on the current information about autistic individuals within the CJS, the authors recognise that there are gaps in our knowledge, a limited number of studies that typically include small sample sizes, and there is an urgent need for further research with this potentially vulnerable population. In particular, there is a need for more precise prevalence rates of autism within the different departments of the CJS, such as contact with police and police custody, court and judicial hearings and prison services. More research is required to establish length of stay within secure settings, rates of recidivism in this population and effects of imprisonment on autistic individuals, and to identify whether autistic individuals are more or less likely to engage in particular types of offending. Many autistic individuals convicted of crimes are never detained in custodial or secure settings and instead are referred to community forensic services or possibly ‘mainstream’ general intellectual disability services. Future research could explore the appropriateness of these referrals and whether these teams and services can meet their needs effectively. Further research is also needed to understand differences between males, females, those who self-report gender incongruence/dysphoria and within specific ethnic backgrounds. Additionally, research into autism within the CJS would benefit from studies exploring the strengths of autistic individuals and how these strengths manifest during contact with the CJS.

The economic consequence of untreated mental health conditions is well known [307]. Although the costs associated with unrecognised and unmet needs of autistic individuals is not known, there are likely to be significant financial implications which should be explored in the future.

The effectiveness of current support strategies is not known. To date, little use has been made of autistic lived experience and expertise in the development of effective approaches and research, which is a significant omission.

## Conclusions

Developments in diagnostic criteria, tools and services have led to increased awareness, recognition and provision of services for autistic individuals, leading to an increase in worldwide autism prevalence rates [308]. Therefore, it is likely that rates of diagnosed autism within the CJS may also increase. Greater attention needs to be given to this potentially vulnerable population and how best to provide support and care as they navigate the CJS.

## Data Availability

Data sharing is not applicable to this article as no datasets were generated or analysed during the current study.

## Appendix 1

**Table 4:** Table of secondary screening tools

**Screening tool**	**Abbreviation**	**Author**	**Age range**	**Self-report**	**Informant report**	**Number of items**	**Administration time in minutes**	**Languages**	**Current access**
Asperger Syndrome Diagnostic Scale	ASDS	[309]	5–18	No	Yes	50	< 15	English and Spanish	Cost
Autism Spectrum Quotient – 50	AQ-50	[53]	16 +	Yes	No	50	10	Multiple	Free
*Autism Spectrum Quotient – 10*	AQ-10	[208]	16 +	Yes	No	10	< 5	Multiple	Free
Autism Spectrum Screening Questionnaire^a^	ASSQ	[310]	6–17	No	Yes	27	10	English and Swedish	Free
*Autism Spectrum Screening Questionnaire – Extended Version*	ASSQ-REV	[311]	6–17	No	Yes	45	N/K	English and Swedish	Free
Childhood Autism Rating Scale, Second Edition	CARS-2	[312]	2 +	No	Yes	15	< 10	English	Cost
Communication Checklist – Adult / Communication Checklist – Self Report	CC-A / CC-SR	[212, 213]	17–79 / 10–80	Yes	Yes	70	< 15	English	Cost
Gilliam Asperger’s Disorder Scale	GADS	[313]	3–22	No	Yes	32	< 10	English	Cost
Gilliam Autism Rating Scale, Third Edition	GARS-3	[314]	3–22	No	Yes	56	< 10	English	Cost
Krug Asperger’s Disorder Index	KADI	[315]	6–22	No	Yes	32	< 20	English	Cost
Ritvo Autism Apserger Diagnostic scale – Revised	RAADS-R	[215]	16 +	Yes	No	80	< 30	English	Free
*Ritvo Autism Apserger Diagnostic scale – 14*	RAADS-14	[214]	16 +	Yes	No	14	< 10	English	Free
Social Communication Questionnaire	SCQ	[203]	2 +	No	Yes	40	10	English	Cost
*Social Communication Questionnaire for Adults with Intellectual Disabilities*^b^	SCQ-AID	[316]	18 +	No	Yes	24	< 10	English	Cost
Social Responsiveness Scale – 2	SRS-2	[211]	19 +	Yes	Yes	65	< 20	English	Cost

This is not an exhaustive list

^a^Can be used with mild intellectual disability

^b^Designed to be used with individuals with intellectual disability

## Appendix 3

**Table 5:** Autistic developmental history assessment tools

***The Autism Diagnostic Interview-Revised (ADI-R)***
To date, the ADI-R has the largest evidence base and highest sensitivity and specificity and is considered the ‘gold standard’ developmental history tool for autism [238, 242]. The ADI-R requires informants who have known the individual since birth and can provide detailed information about childhood (between the ages of 4–5) and current social communication [230, 231]
***The Diagnostic Interview for Social and Communication Disorders (DISCO)***
The DISCO [233] is a validated semi-structured informant interview. It adopts a dimensional approach that provides a clinical description of the individuals strengths and difficulties. This has recently been updated and revised and made considerably shorter
***The Developmental, Dimensional and Diagnostic Interview (3di)***
The 3di [232] is a validated computerised autism interview. It provides the opportunity to include questions about frequently co-occurring conditions such as conduct disorders, ADHD, pragmatic language disorders, Tourette syndrome and obsessive–compulsive disorder. The 3di is suitable for young people aged 4–25 years, but it is not validated for adults over the age of 25
***The Diagnostic Autism Spectrum Interview (DASI)***
The DASI [100], is a semi-structured self- and/or informant interview drawing on the most up-to-date diagnostic manuals (DSM-5 and ICD-11). It is suitable for children (> 2 years), young people and adults and includes prompts for the assessor to consider a range of comorbid conditions. In addition, the DASI includes an observational component which aims to supplement the clinical interview with objective observations of the individual’s social communication strengths and difficulties. Interview questions parallel the diagnostic criteria but, at the time of publication, the tool has yet to be validated
***The Asperger Syndrome and high-functioning autism diagnostic Interview (ASDI)***
The ASDI [234] was developed over several years on the basis of experience with several hundred patients with high-functioning autism spectrum disorders, including a large number with ‘autistic psychopathy’, ‘schizoid personality disorder’ and ‘Asperger syndrome’. It is a 20-item diagnostic interview covering six diagnostic domains; four ‘social’, three ‘interests’, two ‘routines’, five ‘verbal and speech’, five ‘non-verbal communication’ and one ‘motor. At the time of publication, the tool has yet to be validated
***The Autism Clinical Interview for Adults (ACIA)***
The ACIA [235] was adapted from the Family History Interview and refined in accordance with the DSM-5. The ACIA has both subject and informant versions and is comprised of three components. Initially, individuals complete a pre-interview questionnaire. Following this, individuals complete a face-to-face semi-structured interview that covers 22 core items used to calculate social communication and interaction differences and restricted and repetitive behaviours in accordance with the DSM-5 diagnostic criteria for autism spectrum disorder. Finally, the ACIA also includes a co-occurring conditions interview. at the time of publication, the tool has yet to be validated
***Royal College of Psychiatrists Interview Guide for the Diagnostic Assessment of Able Adults with Autism Spectrum Disorder – Revised Edition***
The Royal College of Psychiatrists Interview Guide for the Diagnostic Assessment of Able Adults with Autism Spectrum Disorder – Revised Edition [236] is an interview guide which provides probes to elicit relevant information, support the organisation of this evidence and inform clinical judgment. This tool is designed for use with adults with cognitive abilities within the average range or above

This is not an exhausted list of diagnostic tools to obtain an autism-specific developmental history. However, these tools are considered appropriate for use by consensus. As and when diagnostic criteria for autism changes, decisions as to which tool is most appropriate to use should be revised

## Abbreviations

3Di: The Developmental, Dimensional and Diagnostic Interview
ABAS-3: Adaptive Behavior Assessment System, Third Edition
ACIA: The Autism Clinical Interview for Adults
ADHD: Attention deficit hyperactivity disorder
ADI-R: Autism Diagnostic Interview, Revised
ADOS-2: Autism Diagnostic Observation Schedule, Second Edition
APA/DSM-5-TR: American Psychological Association/Diagnostic and Statistical Manual of Mental Disorders, Fifth Edition, Text Revision
AQ-10: Autism Spectrum Quotient-10
ASDI: The Asperger Syndrome and high-functioning autism diagnostic Interview
CBT: Cognitive behavioural therapy
CC-A: Communication Checklist – Adult
CC-SR: Communication Checklist – Self Report
CHAT: Comprehensive Health Assessment Tool
CJJI: Criminal Justice Joint Inspection
CJS: Criminal Justice System
CPA: Care Programme Approach
CSAAP: Correctional Services Accreditation and Advice Panel
DASI: Diagnostic Autism Spectrum Interview
DISCO: Diagnostic Interview for Social and Communication Disorders
FARAS: Framework for the Assessment of Risk and Protection in Offenders on the Autistic Spectrum
FIND: Forensic Intellectual and Neurodevelopmental Disabilities
FOLS: Forensic Outreach Liaison Services
HCR-20: Historical Clinical and Risk Management – 20
HMI: Her Majesty’s Inspectorate
HMP: Her Majesty’s Prison
HMYOI: Her Majesty’s Young Offenders Institution
NAS: National Autistic Society
NICE: The National Institute for Health and Care Excellence’s
PACE: Police and Criminal Evidence Act 1984
PCL-R: Psychopathy Checklist: Revised
PWI: Personal Wellbeing Index
RAADS-14: Ritvo Autism and Asperger Diagnostic Scale-14
RSVP-V2: Risk of Sexual Violence Protocol, Version 2
SCQ: Social Communication Questionnaire
SOLD: Supporting Offenders with Learning Difficulties
SPELL: Structure, Positive approaches and expectations, Empathy, Low arousal, Links
SPM-2: Sensory Processing Measure, Second Edition
SRS-2: Social Responsiveness Scale, Second Edition
WHO/ICD-11: World Health Organisation/International Classification of Diseases, 11th Revision
YOIs: Youth Offender Institutions

## Footnotes

1 See e.g., *R v BRM* [2022] EWCA Crim 385.

## References

[1] Kenny L, Hattersley C, Molins B, Buckley C, Povey C, Pellicano E (2016). Which terms should be used to describe autism? Perspectives from the UK autism community. Autism.

[2] Bottema-Beutel K, Kapp SK, Lester JN, Sasson NJ, Hand BN (2021). Avoiding ableist language: suggestions for autism researchers. Autism Adulthood.

[3] Keating CT, Hickman L, Leung J, Monk R, Montgomery A, Heath H, Sowden S (2023). Autism-related language preferences of English-speaking individuals across the globe: a mixed methods investigation. Autism Res.

[4] World Health Organization (2019). International Statistical Classification of Diseases and Related Health Problems.

[5] American Psychiatric Association. Diagnostic and statistical manual of mental disorders (5th edn, text revision). Washington, US: APA; 2022.

[6] Hughes JA (2021). Does the heterogeneity of autism undermine the neurodiversity paradigm?. Bioethics.

[7] Brugha TS, Spiers N, Bankart J, Cooper SA, McManus S, Scott FJ, Smith J, Tyrer F (2016). Epidemiology of autism in adults across age groups and ability levels. Br J Psychiatry.

[8] Kogan MD, Vladutiu CJ, Schieve LA, Ghandour RM, Blumberg SJ, Zablotsky B, Perrin JM, Shattuck P, Kuhlthau KA, Harwood RL, Lu MC (2018). The prevalence of parent-reported autism spectrum disorder among US children. Pediatrics..

[9] Matson JL, Kozlowski AM (2011). The increasing prevalence of autism spectrum disorders. Res Autism Spectr Disord.

[10] Maenner MJ, Shaw KA, Bakian AV, Bilder DA, Durkin MS, Esler A, Furnier SM, Hallas L, Hall-Lande J, Hudson A, Hughes MM (2021). Prevalence and characteristics of autism spectrum disorder among children aged 8 years—autism and developmental disabilities monitoring network, 11 sites, United States, 2018. MMWR Surveill Summ.

[11] Loomes R, Hull L, Mandy WP (2017). What is the male-to-female ratio in autism spectrum disorder? A systematic review and meta-analysis. J Am Acad Child Adolesc Psychiatry.

[12] Kallitsounaki A, Williams DM (2023). Autism spectrum disorder and gender dysphoria/incongruence. A systematic literature review and meta-analysis. J Autism Dev Disord..

[13] Matson JL, Horovitz M (2010). Stability of autism spectrum disorders symptoms over time. J Dev Phys Disabil.

[14] LeBlanc LA, Riley AR, Goldsmith TR. Autism spectrum disorders: a lifespan perspective. In.: Clinical assessment and intervention for autism spectrum disorders; 2008. pp. 65–87. Academic Press.

[15] Cashin A, Newman C (2009). Autism in the criminal justice detention system: a review of the literature. J Forensic Nurs.

[16] Collins J, Horton K, Gale-St. Ives E, Murphy G, Barnoux M (2023). A systematic review of autistic people and the criminal justice system: an update of king and murphy (2014). J Autism Dev Disord..

[17] Charles A, Rid A, Davies H, Draper H. Prisoners as research participants: current practice and attitudes in the UK. J Med Ethics. 2014;42(4):246–52. 10.1136/medethics-2012-101059 24958334

[18] Bjørkly S (2009). Risk and dynamics of violence in Asperger’s syndrome: a systematic review of the literature. Aggress Violent Behav.

[19] Browning A, Caulfield L (2011). The prevalence and treatment of people with Asperger’s Syndrome in the criminal justice system. Criminol Crim Justic.

[20] Gunasekaran S, Chaplin E (2012). Autism spectrum disorders and offending. Adv Ment Health Intellect Disabil.

[21] King C, Murphy GH (2014). A systematic review of people with autism spectrum disorder and the criminal justice system. J Autism Dev Disord.

[22] Mouridsen SE, Rich B, Isager T, Nedergaard NJ (2008). Pervasive developmental disorders and criminal behaviour: a case control study. Int J Offender Ther Comp Criminol.

[23] Beadle-Brown J, Richardson L, Guest C, Malovic A, Bradshaw J, Himmerich J (2014). Living in fear: better outcomes for people with learning disabilities and autism.

[24] Sevlever M, Roth ME, Gillis JM (2013). Sexual abuse and offending in autism spectrum disorders. Sex Disabil.

[25] Murphy D (2013). Risk assessment of offenders with an autism spectrum disorder. J Intellect Disabil Offending Behav.

[26] Barry-Walsh JB, Mullen PE (2004). Forensic aspects of Asperger's Syndrome. J Forens Psychiatry Psychol.

[27] Haskins BG, Silva JA (2006). Asperger’s disorder and criminal behavior: forensic-psychiatric considerations. J Am Acad Psychiatry Law.

[28] Allen D, Evans C, Hider A, Hawkins S, Peckett H, Morgan H (2008). Offending behaviour in adults with Asperger syndrome. J Autism Dev Disord.

[29] Woodbury-Smith M, Dein K (2014). Autism spectrum disorder (ASD) and unlawful behaviour: where do we go from here?. J Autism Dev Disord.

[30] Im DS (2016). Template to perpetrate: an update on violence in autism spectrum disorder. Harv Rev Psychiatry.

[31] van der Plas E, Mason D, Happé F. Decision-making in autism: a narrative review. Autism. 2023;27(6):1532–46. 10.1177/13623613221148010 36794463

[32] Woodbury-Smith M, Clare I, Holland AJ, Watson PC, Bambrick M, Kearns A, Staufenberg E (2010). Circumscribed interests and ‘offenders’ with autism spectrum disorders: a case-control study. J Forens Psychiatry Psychol.

[33] Hart-Kerkhoffs LA, Jansen LM, Doreleijers TA, Vermeiren R, Minderaa RB, Hartman CA (2009). Autism spectrum disorder symptoms in juvenile suspects of sex offenses. J Clin Psychiatry.

[34] Kumagami T, Matsuura N (2009). Prevalence of pervasive developmental disorder in juvenile court cases in Japan. J Forens Psychiatry Psychol.

[35] Sutton LR, Hughes TL, Huang A, Lehman C, Paserba D, Talkington V, Taormina R, Walters JB, Fenclau E, Marshall S (2013). Identifying individuals with autism in a state facility for adolescents adjudicated as sexual offenders: a pilot study. Focus Autism Other Dev Disabil.

[36] Allely CS, Creaby-Attwood A (2016). Sexual offending and autism spectrum disorders. J Intellect Disabil Offending Behav.

[37] Payne KL, Maras K, Russell AJ, Brosnan MJ (2020). Self-reported motivations for offending by autistic sexual offenders. Autism.

[38] Brown-Lavoie SM, Viecili MA, Weiss J (2014). Sexual knowledge and victimization in adults with autism spectrum disorders. J Autism Dev Disord.

[39] Dekker LP, Van Der Vegt EJ, Visser K, Tick N, Boudesteijn F, Verhulst FC, Maras A, Greaves-Lord K (2015). Improving psychosexual knowledge in adolescents with autism spectrum disorder: pilot of the tackling teenage training program. J Autism Dev Disord.

[40] Dewinter J, Vermeiren R, Vanwesenbeeck I, Lobbestael J, Van Nieuwenhuizen C (2015). Sexuality in adolescent boys with autism spectrum disorder: self-reported behaviours and attitudes. J Autism Dev Disord.

[41] Murrie DC, Warren JI, Kristiansson M, Dietz PE (2002). Asperger’s syndrome in forensic settings. Int J Forensic Ment Health.

[42] Ledingham R, Mills R (2015). A preliminary study of autism and cybercrime in the context of international law enforcement. Adv Autism.

[43] National Crime Agency. Pathways into Cyber Crime. https://www.nationalcrimeagency.gov.uk/who-we-are/publications/6-pathways-into-cyber-crime-1/file. Accessed 29th Jan 2021.

[44] Payne KL, Russell A, Mills R, Maras K, Rai D, Brosnan M (2019). Is there a relationship between cyber-dependent crime, autistic-like traits and autism?. J Autism Dev Disord.

[45] Higham L, Girardi A, Edwards HV (2021). Clinical and criminal profile of internet offenders with ASD. J Intellect Disabil Offending Behav.

[46] Lim A, Brewer N, Young RL (2023). Revisiting the relationship between cybercrime, autistic traits, and autism. J Autism Dev Disord.

[47] Walter F, Leonard S, Miah S, Shaw J (2021). Characteristics of autism spectrum disorder and susceptibility to radicalisation among young people: A qualitative study. J Forens Psychiatry Psychol.

[48] Långström N, Grann M, Ruchkin V, Sjöstedt G, Fazel S (2009). Risk factors for violent offending in autism spectrum disorder: a national study of hospitalized individuals. J Interpers Violence.

[49] Robinson L, Spencer MD, Thomson LD, Stanfield AC, Owens DG, Hall J, Johnstone EC (2012). Evaluation of a screening instrument for autism spectrum disorders in prisoners. PLoS One..

[50] Chaplin E, McCarthy J. Autism spectrum disorder and offending–a UK perspective. Autism Spectrum Quarterly. 2014(Summer):14–6.

[51] Gunasekaran S (2012). Assessment and management of risk in autism. Adv Ment Health Intellect Disabil.

[52] Westphal A, Allely C. Using structured tools to assess violence risk associated with autism. J Am Acad Psychiatry Law. 2019;47(4): 437–39. 10.29158/JAAPL.003896-19 31744856

[53] Baron-Cohen S, Wheelwright S, Skinner M, Martin J, Clubley E. The Autism-Spectrum Quotient (QA): Evidence from asperger syndrome/high functioning autism, males and females, scientists and mathematicians: Errata. J Autism Dev Disord. 2001;31:5–17. 10.1023/a:1005653411471 11439754

[54] Ashwood KL, Gillan N, Horder J, Hayward H, Woodhouse E, McEwen FS, Findon J, Eklund H, Spain D, Wilson CE, Cadman T (2016). Predicting the diagnosis of autism in adults using the Autism-Spectrum Quotient (AQ) questionnaire. Psychol Med.

[55] Murphy D (2011). Autism Spectrum Quotient (AQ) profiles among male patients within high security psychiatric care: comparison with personality and cognitive functioning. J Forens Psychiatry Psychol.

[56] Woodbury-Smith MR, Clare IC, Holland AJ, Kearns A (2006). High functioning autistic spectrum disorders, offending and other law-breaking: findings from a community sample. J Forens Psychiatry Psychol.

[57] Hippler K, Viding E, Klicpera C, Happé F (2010). Brief report: no increase in criminal convictions in Hans Asperger’s original cohort. J Autism Dev Disord.

[58] Ashworth S (2016). Autism is underdiagnosed in prisoners. BMJ.

[59] Rava J, Shattuck P, Rast J, Roux A (2017). The prevalence and correlates of involvement in the criminal justice system among youth on the autism spectrum. J Autism Dev Disord.

[60] McAdam P (2009). Knowledge and understanding of the autism spectrum amongst prison staff. GAP.

[61] Fazio RL, Pietz CA, Denney RL (2012). An estimate of the prevalence of autism-spectrum disorders in an incarcerated population. Open Access J Forens Psychol.

[62] Murphy D, Bush EL, Puzzo I (2017). Incompatibilities and seclusion of patients with an autism spectrum disorder detained in high-secure psychiatric care. J Intellect Disabil Offending Behav.

[63] Allely CS. Autism spectrum disorder in the criminal justice System: a guide to understanding suspects, defendants and offenders with autism. New York, US: Routledge; 2022.

[64] Bagnall R, Russell A, Brosnan M, Maras K. Autistic adults’ inclination to lie in everyday situations. Autism. 2023;28(3):718–31. 10.1177/13623613231183911 PMC10913365 37572035

[65] Richman KA, Krause-Jensen K, Rodogno R (2022). Autism, the criminal justice system, and transition to adulthood. Transitioning to adulthood with Autism: ethical, legal and social issues.

[66] Slade G. Diverse Perspectives. The challenges for families affected by autism from Black, Asian and Minority Ethinic communities. 2014. Retrieved 30.03.2021. From. https://s3.chorus-mk.thirdlight.com/file/1573224908/63849355948/width=-1/height=-1/format=-1/fit=scale/t=445333/e=never/k=7c17beeb/Diverse-perspectives-report.pdf.

[67] Morgan CN, Roy M, Chance P (2003). Psychiatric comorbidity and medication use in autism: a community survey. BJPsych Bull.

[68] Newman SS, Ghaziuddin M (2008). Violent crime in Asperger syndrome: the role of psychiatric comorbidity. J Autism Dev Disord.

[69] Simonoff E, Pickles A, Charman T, Chandler S, Loucas T, Baird G (2008). Psychiatric disorders in children with autism spectrum disorders: prevalence, comorbidity, and associated factors in a population-derived sample. J Am Acad Child Adolesc Psychiatry.

[70] Joshi G, Wozniak J, Petty C, Martelon MK, Fried R, Bolfek A, Kotte A, Stevens J, Furtak SL, Bourgeois M, Caruso J (2013). Psychiatric comorbidity and functioning in a clinically referred population of adults with autism spectrum disorders: a comparative study. J Autism Dev Disord.

[71] Russell AJ, Murphy CM, Wilson E, Gillan N, Brown C, Robertson DM, Craig MC, Deeley Q, Zinkstok J, Johnston K, McAlonan GM (2016). The mental health of individuals referred for assessment of autism spectrum disorder in adulthood: a clinic report. Autism.

[72] Vermeiren R, Jespers I, Moffitt T (2006). Mental health problems in juvenile justice populations. Child Adolesc Psychiatr Clin N Am.

[73] Hofvander B, Delorme R, Chaste P, Nydén A, Wentz E, Ståhlberg O, Herbrecht E, Stopin A, Anckarsäter H, Gillberg C, Råstam M (2009). Psychiatric and psychosocial problems in adults with normal-intelligence autism spectrum disorders. BMC Psychiatry.

[74] Lugnegård T, Hallerbäck MU, Gillberg C (2011). Psychiatric comorbidity in young adults with a clinical diagnosis of Asperger syndrome. Res Dev Disabil.

[75] Johnston K, Dittner A, Bramham J, Murphy C, Knight A, Russell A (2013). Attention deficit hyperactivity disorder symptoms in adults with autism spectrum disorders. Autism Res.

[76] Sabet J, Underwood L, Chaplin E, Hayward H, McCarthy J (2015). Autism spectrum disorder, attention-deficit hyperactivity disorder and offending. Adv Autism.

[77] Underwood L, McCarthy J, Chaplin E, Forrester A, Mills R, Murphy D (2016). Autism spectrum disorder traits among prisoners. Adv Autism.

[78] Arnevik EA, Helverschou SB (2016). Autism spectrum disorder and co-occurring substance use disorder–A systematic review. Subst Abuse..

[79] Rumball F, Happé F, Grey N (2020). Experience of trauma and PTSD symptoms in autistic adults: risk of PTSD development following DSM-5 and non-DSM-5 traumatic life events. Autism Res.

[80] Hollingdale J, Woodhouse E, Young S, Gudjonsson G, Charman T, Mandy W (2023). Sex differences in conduct and emotional outcomes for young people with hyperactive/inattentive traits and social communication difficulties between 9 and 16 years of age: a growth curve analysis. Psychol Med.

[81] Chaplin E, McCarthy J, Allely CS, Forrester A, Underwood L, Hayward H, Sabet J, Young S, Mills R, Asherson P, Murphy D (2021). Self-harm and Mental Health Characteristics of Prisoners with elevated rates of autistic traits. Res Dev Disabil.

[82] Hwang YI, Srasuebkul P, Foley KR, Arnold S, Trollor JN (2019). Mortality and cause of death of Australians on the autism spectrum. Autism Res.

[83] Kirby AV, Bakian AV, Zhang Y, Bilder DA, Keeshin BR, Coon H (2019). A 20-year study of suicide death in a statewide autism population. Autism Res.

[84] DiBlasi E, Kirby AV, Gaj E, Docherty AR, Keeshin BR, Bakian AV, Coon H (2020). Brief report: genetic links between autism and suicidal behavior—A preliminary investigation. J Autism Dev Disord.

[85] McDonnell CG, DeLucia EA, Hayden EP, Anagnostou E, Nicolson R, Kelley E, Georgiades S, Liu X, Stevenson RA (2020). An exploratory analysis of predictors of youth suicide-related behaviors in autism spectrum disorder: implications for prevention science. J Autism Dev Disord.

[86] Crane L, Adams F, Harper G, Welch J, Pellicano E (2019). ‘Something needs to change’: mental health experiences of young autistic adults in England. Autism.

[87] Camm-Crosbie L, Bradley L, Shaw R, Baron-Cohen S, Cassidy S (2019). ‘People like me don’t get support’: Autistic adults’ experiences of support and treatment for mental health difficulties, self-injury and suicidality. Autism.

[88] Costa AP, Loor C, Steffgen G (2020). Suicidality in adults with autism spectrum disorder: the role of depressive symptomatology, alexithymia, and antidepressants. J Autism Dev Disord.

[89] Arwert TG, Sizoo BB (2020). Self-reported suicidality in male and female adults with autism spectrum disorders: rumination and self-esteem. J Autism Dev Disord.

[90] Cassidy S, Bradley L, Shaw R, Baron-Cohen S (2018). Risk markers for suicidality in autistic adults. Mol Autism.

[91] HMI PROBATION. A joint inspection of the treatment of offenders with learning disabilities, within the criminal justice system: phase one – from arrest to sentence. In: PROBATION, H. M. S. I. O. (ed.). Manchester: Her Majesty's Inspectorate of Probation; 2014.

[92] HMI PROBATION. A joint inspection of the treatment of offenders with learning disabilities, within the criminal justice system: phase two – in custody and the community. In: PROBATION, H. M. S. I. O. (ed.). Manchester: Her Majesty's Inspectorate of Probation; 2015.

[93] Slavny-Cross R, Allison C, Griffiths S, Baron-Cohen S (2023). Are autistic people disadvantaged by the criminal justice system? A case comparison. Autism.

[94] Criminal Justice Joint Inspection. Neurodiversity in the criminal justice system: A review of the evidence. Neurodiversity in the criminal justice system: a review of evidence (justiceinspectorates.gov.uk). 2021. Assessed 23rd Dec 2021.

[95] Salerno AC, Schuller RA (2019). A mixed-methods study of police experiences of adults with autism spectrum disorder in Canada. Int J Law Psychiatry.

[96] Tint A, Palucka AM, Bradley E, Weiss JA, Lunsky Y (2017). Correlates of police involvement among adolescents and adults with autism spectrum disorder. J Autism Dev Disord.

[97] Crane L, Maras KL, Hawken T, Mulcahy S, Memon A (2016). Experiences of autism spectrum disorder and policing in England and Wales: Surveying police and the autism community. J Autism Dev Disord.

[98] Maras K. Obtaining testimony from autistic people. Handbook of Autism Spectrum Disorder and the Law. 2021. p. 145–83.

[99] Bagnall R, Cadman A, Russell A, Brosnan M, Otte M, Maras KL (2023). Police suspect interviews with autistic adults: the impact of truth telling versus deception on testimony. Front Psychol.

[100] Young RL, Brewer N (2020). Brief report: perspective taking deficits, autism spectrum disorder, and allaying police officers’ suspicions about criminal involvement. J Autism Dev Disord.

[101] Home Office. Police and Criminal Evidence Act 1984 (PACE) – Code C Revised. Code of Practice for the detention, treatment and questioning of persons by Police Officers. 2019. https://assets.publishing.service.gov.uk/government/uploads/system/uploads/attachment_data/file/903473/pace-code-c-2019.pdf.

[102] Young S, Goodwin EJ, Sedgwick O, Gudjonsson GH (2013). The effectiveness of police custody assessments in identifying suspects with intellectual disabilities and attention deficit hyperactivity disorder. BMC Med.

[103] Hepworth D (2017). A critical review of current police training and policy for autism spectrum disorder. J Intellect Disabil Offending Behav.

[104] Maras KL, Crane L, Mulcahy S, Hawken T, Cooper P, Wurtzel D, Memon A (2017). Brief report: Autism in the courtroom: Experiences of legal professionals and the autism community. J Autism Dev Disord.

[105] Gibbs V, Haas K (2020). Interactions between the police and the autistic community in Australia: experiences and perspectives of autistic adults and parents/carers. J Autism Dev Disord.

[106] Haas K, Gibbs V (2021). Does a person’s autism play a role in their interactions with police: the perceptions of autistic adults and parent/carers. J Autism Dev Disord.

[107] Holloway CA, Munro N, Jackson J, Phillips S, Ropar D (2020). Exploring the autistic and police perspectives of the custody process through a participative walkthrough. Res Dev Disabil.

[108] North AS, Russell AJ, Gudjonsson GH (2008). High functioning autism spectrum disorders: an investigation of psychological vulnerabilities during interrogative interview. J Forens Psychiatry Psychol.

[109] Salseda LM, Dixon DR, Fass T, Miora D, Leark RA (2011). An evaluation of Miranda rights and interrogation in autism spectrum disorders. Res Autism Spectr Disord.

[110] Debbaudt D, Rothman D (2001). Contact with individuals with autism: Effective resolutions. FBI L Enforcement Bull.

[111] Murphy D (2018). Interviewing individuals with an autism spectrum disorder in forensic settings. Int J Forensic Ment Health.

[112] Lim A, Young RL, Brewer N (2022). Autistic adults may be erroneously perceived as deceptive and lacking credibility. J Autism Dev Disord.

[113] Gudjonsson GH. The psychology of false confessions: forty years of science and practice. Hoboken: Wiley; 2018.

[114] Gudjonsson GH, Young S, Bramham J (2007). Interrogative suggestibility in adults diagnosed with attention-deficit hyperactivity disorder (ADHD). A potential vulnerability during police questioning. Pers Individ Dif..

[115] Gudjonsson G, Young S (2021). An investigation of ‘don't know’ and ‘direct explanation’ response styles on the Gudjonsson Suggestibility Scale: a comparison of three different vulnerable adult groups. Pers Individ Dif.

[116] Gudjonsson GH, Sigurdsson JF, Sigfusdottir ID, Young S (2012). False confessions to police and their relationship with conduct disorder, ADHD, and life adversity. Pers Individ Dif.

[117] Gudjonsson GH, Sigurdsson JF, Sigfusdottir ID, Asgeirsdottir BB, González RA, Young S (2016). A national epidemiological study investigating risk factors for police interrogation and false confession among juveniles and young persons. Soc Psychiatry Psychiatr Epidemiol.

[118] Maras K, Bowler DM (2011). Brief report: schema consistent misinformation effects in eyewitnesses with autism spectrum disorder. J Autism Dev Disord.

[119] Maras KL, Bowler DM (2012). Brief report: suggestibility, compliance and psychological traits in high-functioning adults with autism spectrum disorder. Res Autism Spectr Disord.

[120] Chandler RJ, Russell A, Maras KL (2019). Compliance in autism: self-report in action. Autism.

[121] Maras KL, Memon A, Lambrechts A, Bowler DM (2013). Recall of a live and personally experienced eyewitness event by adults with autism spectrum disorder. J Autism Dev Disord.

[122] Maras KL, Mulcahy S, Memon A, Picariello F, Bowler DM (2014). Evaluating the effectiveness of the self-administered interview© for witnesses with autism spectrum disorder. Appl Cogn Psychol.

[123] Maras K, Norris JE, Nicholson J, Heasman B, Remington A, Crane L (2021). Ameliorating the disadvantage for autistic job seekers: an initial evaluation of adapted employment interview questions. Autism.

[124] Cooper P, Mattison M (2017). Intermediaries, vulnerable people and the quality of evidence: an international comparison of three versions of the English intermediary model. Int J Evid Proof.

[125] Home Office. Youth Justice and Criminal Evidence Act 1999; 2023. https://www.legislation.gov.uk/ukpga/1999/23/contents.

[126] Debbaudt D. Autism, advocates, and law enforcement professionals: Recognizing and reducing risk situations for people with autism spectrum disorders. London: Jessica Kingsley Publishers; 2001.

[127] Allely CS, Cooper P. Jurors’ and judges’ evaluation of defendants with autism and the impact on sentencing: a systematic Preferred Reporting Items for Systematic Reviews and Meta-analyses (PRISMA) review of autism spectrum disorder in the courtroom. J Law Med. 2017;25(1):105–23. 29978627

[128] Cooper P, Allely C (2017). You can’t judge a book by its cover: evolving professional responsibilities, liabilities and'judgecraft'when a party has Asperger's Syndrome. N Ir Legal Q.

[129] Maras K, Marshall I, Sands C (2019). Mock juror perceptions of credibility and culpability in an autistic defendant. J Autism Dev Disord.

[130] Berryessa CM (2020). Defendants with autism spectrum disorder in criminal court: a judges’ toolkit. Drexel L Rev.

[131] Berryessa CM (2014). Judiciary views on criminal behaviour and intention of offenders with high-functioning autism. J Intellect Disabil Offending Behav.

[132] Berryessa CM (2014). Judicial perceptions of media portrayals of offenders with high functioning autistic spectrum disorders. Int J Criminol Sociol.

[133] Caliman CR, Berryessa CM (2023). Legal decision-makers in criminal cases involving autism spectrum disorder: a review of the research and a call for action.

[134] Freckelton I (2013). Autism spectrum disorder: forensic issues and challenges for mental health professionals and courts. J Appl Res Intellect Disabil.

[135] Berryessa CM, Milner LC, Garrison NA, Cho MK (2015). Impact of psychiatric information on potential jurors in evaluating high-functioning autism spectrum disorder (hfASD). J Ment Health Res Intellect Disabil.

[136] Berryessa CM (2016). Brief report: judicial attitudes regarding the sentencing of offenders with high functioning autism. J Autism Dev Disord.

[137] Slavny-Cross R, Allison C, Griffiths S, Baron-Cohen S (2022). Autism and the criminal justice system: an analysis of 93 cases. Autism Res.

[138] Cea CN (2014). Autism and the criminal defendant. Johns L Rev.

[139] Berryessa CM (2017). Educator of the court: the role of the expert witness in cases involving autism spectrum disorder. Psychol Crime Law.

[140] Gerry QC. Trauma-informed courts (Pt 1). New Law J. 171(7919):16.

[141] Gerry QC. Trauma-informed courts (Pt 2). New Law J. 171(7922):16.

[142] Van der Kolk BA (1994). The body keeps the score: memory and the evolving psychobiology of posttraumatic stress. Harv Rev Psychiatry.

[143] Brewer RJ, Davies GM, Blackwood NJ (2016). Fitness to plead: the impact of autism spectrum disorder. J Forens Psychol Pract.

[144] Freckelton Sc I, List D (2009). Asperger’s disorder, criminal responsibility and criminal culpability. Psychiatry Psychol Law.

[145] O’Sullivan OP (2018). Autism spectrum disorder and criminal responsibility: historical perspectives, clinical challenges and broader considerations within the criminal justice system. Ir J Psychol Med.

[146] Grant T, Furlano R, Hall L, Kelley E (2018). Criminal responsibility in autism spectrum disorder: a critical review examining empathy and moral reasoning. Can Psychol Can.

[147] Michna I, Trestman R (2016). Correctional management and treatment of autism spectrum disorder. J Am Acad Psychiatry Law.

[148] de la Cuesta G (2010). A selective review of offending behaviour in individuals with autism spectrum disorders. J Intellect Disabil Offending Behav.

[149] Van Roekel E, Scholte RH, Didden R (2010). Bullying among adolescents with autism spectrum disorders: Prevalence and perception. J Autism Dev Disord.

[150] Lewis A, Foster M, Hughes C, Turner K (2016). Management of prisoners with autism is not perfect but is improving. BMJ.

[151] Newman C, Cashin A, Graham I (2019). Identification of service development needs for incarcerated adults with autism spectrum disorders in an Australian prison system. Int J Prison Health.

[152] Vinter LP, Dillon G, Winder B (2023). ‘People don’t like you when you’re different’: exploring the prison experiences of autistic individuals. Psychol Crime Law.

[153] Murphy D, Mullens H (2017). Examining the experiences and quality of life of patients with an autism spectrum disorder detained in high secure psychiatric care. Adv Autism.

[154] Helverschou SB, Steindal K, Nøttestad JA, Howlin P (2018). Personal experiences of the criminal justice system by individuals with autism spectrum disorders. Autism.

[155] Allely CS (2015). Experiences of prison inmates with autism spectrum disorders and the knowledge and understanding of the spectrum amongst prison staff: a review. J Intellect Disabil Offending Behav.

[156] Vinter LP (2020). Working with autistic individuals in prison-based interventions to address sexual offending.

[157] English K, Heil P (2005). Prison rape: what we know today. Correct Compend.

[158] Dein K, Woodbury-Smith M (2010). Asperger syndrome and criminal behaviour. Adv Psychiatr Treat.

[159] Lewis A, Pritchett R, Hughes C, Turner K (2015). Development and implementation of autism standards for prisons. J Intellect Disabil Offending Behav.

[160] Frith U, Hill E, editors. Autism: Mind and brain. Oxford: Oxford University Press; 2004.

[161] Haq I, Le Couteur A (2004). Autism spectrum disorder. Medicine.

[162] Freckelton I (2020). Autism spectrum disorder and suitability for extradition: love v the Government of the United States [2018] 1 WLR 2889;[2018] EWHC 172 (Admin) per Burnett LCJ and Ouseley. J Psychiatry Psychol Law.

[163] Freckelton I. Expert evidence about autism spectrum disorder. Handbook of Autism Spectrum Disorder and the Law. 2021. p. 39–69.

[164] Robertson CE, McGillivray JA (2015). Autism behind bars: a review of the research literature and discussion of key issues. J Forens Psychiatry Psychol.

[165] Griffin-Shelley E (2010). An Asperger's adolescent sex addict, sex offender: a case study. Sex Addict Compuls.

[166] Murphy D (2010). Understanding offenders with autism-spectrum disorders: what can forensic services do?: Commentary on… Asperger Syndrome and Criminal Behaviour. Adv Psychiatr Treat.

[167] Melvin CL, Langdon PE, Murphy GH (2017). Treatment effectiveness for offenders with autism spectrum conditions: a systematic review. Psychol Crime Law.

[168] Hill E, Berthoz S, Frith U (2004). Brief report: cognitive processing of own emotions in individuals with autistic spectrum disorder and in their relatives. J Autism Dev Disord.

[169] Lombardo MV, Barnes JL, Wheelwright SJ, Baron-Cohen S (2007). Self-referential cognition and empathy in autism. PLoS One.

[170] Berthoz S, Hill EL (2005). The validity of using self-reports to assess emotion regulation abilities in adults with autism spectrum disorder. Eur Psychiatry.

[171] National Institute for Health and Care Excellence (NICE). Autism spectrum disorder in adults: diagnosis and management. Updated 18th Aug 2016. https://www.nice.org.uk/guidance/CG142/chapter/1-Guidance#identification-and-assessment. 32186834

[172] National Institute for Health and Care Excellence (NICE). Updated 20^th^ December 2017. Autism spectrum disorder in under 19s: recognition, referral and diagnosis. https://www.nice.org.uk/guidance/cg128/chapter/Recommendations#autism-diagnostic-assessment-for-children-and-young-people. Accessed 29th Jan 2023. 31999412

[173] Young S, Gudjonsson G, Chitsabesan P, Colley B, Farrag E, Forrester A, Hollingdale J, Kim K, Lewis A, Maginn S, Mason P (2018). Identification and treatment of offenders with attention-deficit/hyperactivity disorder in the prison population: a practical approach based upon expert consensus. BMC Psychiatry.

[174] Young S, Hollingdale J, Absoud M, Bolton P, Branney P, Colley W, Craze E, Dave M, Deeley Q, Farrag E, Gudjonsson G (2020). Guidance for identification and treatment of individuals with attention deficit/hyperactivity disorder and autism spectrum disorder based upon expert consensus. BMC Med.

[175] Gillespie-Smith K, Ballantyne C, Branigan HP, Turk DJ, Cunningham SJ (2018). The I in autism: severity and social functioning in autism are related to self-processing. Br J Dev Psychol.

[176] Lee V, Duku E, Zwaigenbaum L, Bennett T, Szatmari P, Elsabbagh M, Kerns C, Mirenda P, Smith IM, Ungar WJ, Vaillancourt T (2020). Temperament influences the relationship between symptom severity and adaptive functioning in children with autism spectrum disorder. Autism.

[177] Nylander L, Holmqvist M, Gustafson L, Gillberg C (2013). Attention-deficit/hyperactivity disorder (ADHD) and autism spectrum disorder (ASD) in adult psychiatry. A 20-year register study. Nord J Psychiatry..

[178] Aggarwal S, Angus B (2015). Misdiagnosis versus missed diagnosis: diagnosing autism spectrum disorder in adolescents. Australas Psychiatry.

[179] Takara K, Kondo T, Kuba T. How and why is autism spectrum disorder misdiagnosed in autism. Autism. 2015;15:16

[180] Van Schalkwyk GI, Peluso F, Qayyum Z, McPartland JC, Volkmar FR (2015). Varieties of misdiagnosis in ASD: an illustrative case series. J Autism Dev Disord.

[181] Fusar-Poli L, Brondino N, Politi P, Aguglia E (2022). Missed diagnoses and misdiagnoses of adults with autism spectrum disorder. Eur Arch Psychiatry Clin Neurosci.

[182] Gordon C, Lewis M, Knight D, Salter E. Differentiating between borderline personality disorder and autism spectrum disorder. Ment Health Pract. 2020;23(3).

[183] Cook ML, Zhang Y, Constantino JN (2020). On the continuity between autistic and schizoid personality disorder trait burden: a prospective study in adolescence. J Nerv Ment Dis.

[184] Carthy E, Murphy D (2021). Comorbid autism spectrum disorder and antisocial personality disorder in forensic settings. J Am Acad Psychiatry Law.

[185] Hare RD (2003). Psychopathy checklist—revised (PCL-R) Technical Manual Psychological Assessment.

[186] Murphy D (2007). Hare psychopathy checklist revised profiles of male patients with Asperger’s syndrome detained in high security psychiatric care. J Forens Psychiatry Psychol.

[187] Allely CS (2019). Understanding and recognising the female phenotype of autism spectrum disorder and the “camouflage” hypothesis: a systematic PRISMA review. Adv Autism.

[188] Fombonne E (2020). Camouflage and autism. J Child Psychol Psychiatry.

[189] Suckle EK (2021). DSM-5 and challenges to female autism identification. J Autism Dev Disord.

[190] Kreiser NL, White SW (2014). ASD in females: are we overstating the gender difference in diagnosis?. Clin Child Fam Psychol Rev.

[191] Schuck RK, Flores RE, Fung LK (2019). Brief report: sex/gender differences in symptomology and camouflaging in adults with autism spectrum disorder. J Autism Dev Disord.

[192] Mandy W, Chilvers R, Chowdhury U, Salter G, Seigal A, Skuse D (2012). Sex differences in autism spectrum disorder: evidence from a large sample of children and adolescents. J Autism Dev Disord.

[193] Allely C (2019). Exploring the female autism phenotype of repetitive behaviours and restricted interests (RBRIs): a systematic PRISMA review. Adv Autism.

[194] Cook J, Hull L, Crane L, Mandy W (2021). Camouflaging in autism: a systematic review. Clin Psychol Rev.

[195] Department of Health and social care. The Bamford Review of mental health and learning disability (Northern Ireland) Forensic Services. 2006. Accessed 29th March 2022. https://www.health-ni.gov.uk/sites/default/files/publications/dhssps/Forensic%20Services%20Report.pdf.

[196] Railey KS, Love AM, Campbell JM (2021). A scoping review of autism spectrum disorder and the criminal justice system. Rev J Autism Dev Disord.

[197] Al-Attar Z. Development and evaluation of guidance to aid risk assessments of offenders with autism. [MA Dissertation]. Sheffield: Sheffield Hallam University; 2018.

[198] Al-Attar Z. Introducing the FARAS—A framework to aid risk assessment with offenders on the autistic spectrum. Innational autistic society’s 18th international conference on offenders with an intellectual and/or developmental disability, Birmingham. 2019.

[199] Beadle-Brown J, Mills R (2018). Understanding and responding to autism: the SPELL framework.

[200] Murphy D, Broyd JG (2020). Evaluation of autism awareness training provided to staff working in a high secure psychiatric care hospital. Adv Autism.

[201] Department of Health and social care. ‘Right to be heard’: The Government’s response to the consultation on learning disability and autism training for health and care staff. 2019. Accessed 8^th^ Jan 2022. https://assets.publishing.service.gov.uk/government/uploads/system/uploads/attachment_data/file/844356/autism-and-learning-disability-training-for-staff-consultation-response.pdf.

[202] Chitsabesan P, Lennox C, Theodosiou L, Law H, Bailey S, Shaw J (2014). The development of the comprehensive health assessment tool for young offenders within the secure estate. J Forens Psychiatry Psychol.

[203] Rutter M (2003). Social communication questionnaire (SCQ).

[204] Chandler S, Charman T, Baird G, Simonoff E, Loucas TO, Meldrum D, Scott M, Pickles A (2007). Validation of the social communication questionnaire in a population cohort of children with autism spectrum disorders. J Am Acad Child Adolesc Psychiatry.

[205] Bölte S, Holtmann M, Poustka F. The Social Communication Questionnaire (SCQ) as a screener for autism spectrum disorders: additional evidence and cross-cultural validity. J Am Acad Child Adolesc Psychiatry. 2008;47(6):719–20. 10.1097/CHI.0b013e31816c42bd 18496329

[206] Hollingdale J, Woodhouse E, Young S, Fridman A, Mandy W (2020). Autistic spectrum disorder symptoms in children and adolescents with attention-deficit/hyperactivity disorder: a meta-analytical review. Psychol Med.

[207] Cummins RA, Lau A (2005). Personal wellbeing index–school children.

[208] Allison C, Auyeung B, Baron-Cohen S (2012). Toward brief “red flags” for autism screening: the short autism spectrum quotient and the short quantitative checklist in 1,000 cases and 3,000 controls. J Am Acad Child Adolesc Psychiatry.

[209] National Institute for Health and Care Excellence (NICE). Autism spectrum disorder in adults: diagnosis and management. Updated 14^th^ June 2021. https://www.nice.org.uk/guidance/cg142/resources/autism-spectrum-quotient-aq10-test-143968. 32186834

[210] McCarthy J, Chaplin E, Underwood L, Forrester A, Hayward H, Sabet J, Young S, Asherson P, Mills R, Murphy D (2015). Screening and diagnostic assessment of neurodevelopmental disorders in a male prison. J Intellect Disabil Offending Behav.

[211] Constantino JN, Gruber CP (2012). Social responsiveness scale: SRS-2.

[212] Whitehouse AO (2009). The Communication Checklist-Adult version (CC-A).

[213] Bishop DV, Whitehouse AJ, Sharp M (2009). The Communication Checklist-Self Report (CC-SR).

[214] Eriksson JM, Andersen LM, Bejerot S (2013). RAADS-14 Screen: validity of a screening tool for autism spectrum disorder in an adult psychiatric population. Mol Autism.

[215] Ritvo RA, Ritvo ER, Guthrie D, Ritvo MJ, Hufnagel DH, McMahon W, Tonge B, Mataix-Cols D, Jassi A, Attwood T, Eloff J (2011). The Ritvo Autism Asperger Diagnostic Scale-Revised (RAADS-R): a scale to assist the diagnosis of autism spectrum disorder in adults: an international validation study. J Autism Dev Disord.

[216] Moloney N, Gulati G (2022). Autism spectrum disorder and Irish prisoners. Ir J Psychol Med.

[217] Jones SL, Johnson M, Alty B, Adamou M (2021). The effectiveness of RAADS-R as a screening tool for adult ASD populations. Autism Res Treat..

[218] Alnazly EK, Abojedi A (2019). Psychological distress and perceived burden in caregivers of persons with autism spectrum disorder. Perspect Psychiatr Care.

[219] Baykal S, Karakurt MN, Çakır M, Karabekiroğlu K (2019). An examination of the relations between symptom distributions in children diagnosed with autism and caregiver burden, anxiety and depression levels. Community Ment Health J.

[220] Marsack-Topolewski CN, Maragakis A (2021). Relationship between symptom severity and caregiver burden experienced by parents of adults with autism. Focus Autism Other Dev Disabil.

[221] World Health Organization. The ICD-10 classification of mental and behavioural disorders: diagnostic criteria for research. World Health Organization: WHO; 1993.

[222] Milton DE (2014). Autistic expertise: a critical reflection on the production of knowledge in autism studies. Autism.

[223] Pugliese CE, Anthony L, Strang JF, Dudley K, Wallace GL, Kenworthy L (2015). Increasing adaptive behavior skill deficits from childhood to adolescence in autism spectrum disorder: role of executive function. J Autism Dev Disord.

[224] Kraper CK, Kenworthy L, Popal H, Martin A, Wallace GL (2017). The gap between adaptive behavior and intelligence in autism persists into young adulthood and is linked to psychiatric co-morbidities. J Autism Dev Disord.

[225] McQuaid GA, Pelphrey KA, Bookheimer SY, Dapretto M, Webb SJ, Bernier RA, McPartland JC, Van Horn JD, Wallace GL (2021). The gap between IQ and adaptive functioning in autism spectrum disorder: Disentangling diagnostic and sex differences. Autism.

[226] Harrison PL, Oakland T. ABAS-3. Torrance: Western Psychological Services; 2015.

[227] Parham LD, Ecker CL, Kuhaneck HM, Henry DA, Glennon TJ. Sensory Processing Measure, (SPM-2). Torrance: Western Psychological Services. 2021.

[228] Lai MC, Kassee C, Besney R, Bonato S, Hull L, Mandy W, Szatmari P, Ameis SH (2019). Prevalence of co-occurring mental health diagnoses in the autism population: a systematic review and meta-analysis. Lancet Psychiatry.

[229] Lord C, Brugha TS, Charman T, Cusack J, Dumas G, Frazier T, Jones EJ, Jones RM, Pickles A, State MW, Taylor JL (2020). Autism spectrum disorder. Nat Rev Dis Primers.

[230] Rutter M, Le Couteur A, Lord C. Autism diagnostic interview-revised. Los Angeles, CA: Western Psychological Services. 2003;29(2003):30.

[231] Lord C, Rutter M, Le Couteur A (1994). Autism diagnostic interview-revised: a revised version of a diagnostic interview for caregivers of individuals with possible pervasive developmental disorders. J Autism Dev Disord.

[232] Skuse D, Warrington R, Bishop D, Chowdhury U, Lau J, Mandy W, Place M (2004). The developmental, dimensional and diagnostic interview (3di): a novel computerized assessment for autism spectrum disorders. J Am Acad Child Adolesc Psychiatry.

[233] Wing L, Leekam SR, Libby SJ, Gould J, Larcombe M (2002). The diagnostic interview for social and communication disorders: Background, inter-rater reliability and clinical use. J Child Psychol Psychiatry.

[234] Gillberg C, Gillberg C, Råstam M, Wentz E (2001). The Asperger Syndrome (and high-functioning autism) Diagnostic Interview (ASDI): a preliminary study of a new structured clinical interview. Autism.

[235] Wigham S, Ingham B, Le Couteur A, Berney T, Ensum I, Parr JR (2020). Development and initial utility of the Autism Clinical Interview for Adults: a new adult autism diagnostic measure. Autism Adulthood.

[236] Berney T, Brugha T, Carpenter P. Royal College of Psychiatrists Interview Guide for the Diagnostic Assessment of Able Adults with Autism Spectrum Disorder (ASD). 2017. https://www.rcpsych.ac.uk/docs/default-source/members/sigs/neurodevelopmental-psychiatry-special-interest-group-ndpsig/ndpsig-autism-diagnostic-interview-guide-2.pdf?sfvrsn=1dc6557_2.

[237] Le Couteur A, Haden G, Hammal D, McConachie H (2008). Diagnosing autism spectrum disorders in pre-school children using two standardised assessment instruments: the ADI-R and the ADOS. J Autism Dev Disord.

[238] Falkmer T, Anderson K, Falkmer M, Horlin C (2013). Diagnostic procedures in autism spectrum disorders: a systematic literature review. Eur Child Adolesc Psychiatry.

[239] Woolfenden S, Sarkozy V, Ridley G, Williams K (2012). A systematic review of the diagnostic stability of autism spectrum disorder. Res Autism Spectr Disord.

[240] Lord C, Luyster RJ, Gotham K, Guthrie W (2012). Edition (ADOS-2) Manual (Part II). Toddler module (Autism Diagnostic Observation Schedule, Second).

[241] Lord C, Rutter M, DiLavore PC, Risi S, Gotham K, Bishop S (2012). Edition (ADOS-2) Manual (Part I). Modules 1–4 (Autism Diagnostic Observation Schedule, Second).

[242] Kanne SM, Randolph JK, Farmer JE (2008). Diagnostic and assessment findings: a bridge to academic planning for children with autism spectrum disorders. Neuropsychol Review.

[243] Webster CD, Douglas KS, Eaves D, Hart SD. HCR-20: assessing risk for violence version 2. Vancouver: Simon Fraser University; 1997.

[244] Douglas KS, Hart SD, Webster CD, Belfrage H (2013). HCR-20V3: Assessing risk for violence: User guide.

[245] Shine J, Cooper-Evans S (2016). Developing an autism specific framework for forensic case formulation. J Intellect Disabil Offending Behav.

[246] Sugrue DP. Forensic assessment of individuals with autism spectrum charged with child pornography violations. Caught in the web of the criminal justice system: autism, developmental disabilities, and sex offenses. 2017. p. 112-39.

[247] Girardi A, Hancock-Johnson E, Thomas C, Wallang PM (2019). Assessing the risk of inpatient violence in autism spectrum disorder. J Am Acad Psychiatry Law.

[248] Allely CS, Dubin L (2018). The contributory role of autism symptomology in child pornography offending: why there is an urgent need for empirical research in this area. J Intellect Disabil Offending Behav.

[249] Hart SD, Kropp PR, Laws DR, Klaver J, Logan C, Watt KA (2022). The risk for sexual violence protocol (RSVP): Structured professional guidelines for assessing risk of sexual violence. Version 2.

[250] Frith U, Happé F (2005). Autism spectrum disorder. Curr Biol.

[251] Dewey M, Frith U (1991). Living with Asperger’s syndrome. Autism and Asperger Syndrome.

[252] Howlin P, Alcock J, Burkin C (2005). An 8 year follow-up of a specialist supported employment service for high-ability adults with autism or Asperger syndrome. Autism.

[253] Martin N, Milton DE, Sims T, Dawkins G, Baron-Cohen S, Mills R (2017). Does, “mentoring” offer effective support to autistic adults? A mixed-methods pilot study. Adv Autism.

[254] Lord C, Wagner A, Rogers S, Szatmari P, Aman M, Charman T, Dawson G, Durand VM, Grossman L, Guthrie D, Harris S (2005). Challenges in evaluating psychosocial interventions for autistic spectrum disorders. J Autism Dev Disord.

[255] Seida JK, Ospina MB, Karkhaneh M, Hartling L, Smith V, Clark B (2009). Systematic reviews of psychosocial interventions for autism: an umbrella review. Dev Med Child Neurol.

[256] Jutel A, Nettleton S (2011). Towards a sociology of diagnosis: reflections and opportunities. Soc Sci Med.

[257] Taylor JL, McPheeters ML, Sathe NA, Dove D, Veenstra-VanderWeele J, Warren Z (2012). A systematic review of vocational interventions for young adults with autism spectrum disorders. Pediatrics.

[258] Bishop-Fitzpatrick L, Minshew NJ, Eack SM. A systematic review of psychosocial interventions for adults with autism spectrum disorders. Adolescents and adults with autism spectrum disorders. 2014. p. 315-27. 10.1007/s10803-012-1615-8 PMC3508309 22825929

[259] Ozsivadjian A, Knott F (2011). Anxiety problems in young people with autism spectrum disorder: a case series. Clin Child Psychol Psychiatry.

[260] Wang X, Zhao J, Huang S, Chen S, Zhou T, Li Q, Luo X, Hao Y (2021). Cognitive behavioral therapy for autism spectrum disorders: a systematic review. Pediatrics..

[261] Hare DJ. Developing psychotherapeutic interventions for people with autism spectrum disorders. Psychological therapies for adults with intellectual disabilities. 2013. p. 193-206.

[262] Taylor JL, Novaco RW, Gillmer B, Thorne I (2002). Cognitive–behavioural treatment of anger intensity among offenders with intellectual disabilities. J Appl Res Intellect Disabil.

[263] Taylor JL, Novaco RW, Gillmer BT, Robertson A, Thorne I (2005). Individual cognitive-behavioural anger treatment for people with mild-borderline intellectual disabilities and histories of aggression: a controlled trial. Br J Clin Psychol.

[264] Lindsay WR, Steele L, Smith AH, Quinn K, Allan R (2006). A community forensic intellectual disability service: twelve year follow up of referrals, analysis of referral patterns and assessment of harm reduction. Leg Criminol Psychol.

[265] Keen D, Webster A, Ridley G (2016). How well are children with autism spectrum disorder doing academically at school? An overview of the literature. Autism.

[266] Visher CA, Lattimore PK (2007). Major study examines prisoners and their re-entry needs. NIJ J.

[267] Ramakers A, Nieuwbeerta P, Van Wilsem J, Dirkzwager A (2017). Not just any job will do: a study on employment characteristics and recidivism risks after release. I J Offender Ther Comp Criminol.

[268] Office for National Statistics (ONS). ONS website, Outcomes for disabled people in the UK: 2021, release date 10^th^ February 2022, correction 7^th^ June 2022.

[269] Doyle N, McDowall A (2021). Diamond in the rough? An “empty review” of research into “neurodiversity” and a road map for developing the inclusion agenda. Equal Divers Incl.

[270] Scott M, Milbourn B, Falkmer M, Black M, Bӧlte S, Halladay A, Lerner M, Taylor JL, Girdler S (2019). Factors impacting employment for people with autism spectrum disorder: a scoping review. Autism.

[271] Ezerins ME, Simon LS, Vogus TJ, Gabriel AS, Calderwood C, Rosen CC. Autism and employment: a review of the “new frontier” of diversity research. J Manage. 2023; 0(0).

[272] Helverschou SB, Rasmussen K, Steindal K, Søndanaa E, Nilsson B, Nøttestad JA (2015). Offending profiles of individuals with autism spectrum disorder: a study of all individuals with autism spectrum disorder examined by the forensic psychiatric service in Norway between 2000 and 2010. Autism.

[273] O’Brien GR, Bell G (2004). Learning disability, autism and offending behaviour. Adolesc Forensic Psychiatry.

[274] Dockrell JE, Lindsay G, Palikara O (2011). Explaining the academic achievement at school leaving for pupils with a history of language impairment: previous academic achievement and literacy skills. Child Lang Teach Ther.

[275] Bryan K, Freer J, Furlong C (2007). Language and communication difficulties in juvenile offenders. Int J Lang Comm Disord.

[276] Snow PC, Powell MB (2008). Oral language competence, social skills and high-risk boys: what are juvenile offenders trying to tell us?. Child Soc.

[277] Mouridsen SE, Hauschild KM (2009). A long-term study of offending in individuals diagnosed with a developmental language disorder as children. Int J Speech Lang Pathol.

[278] Bercow J. The Bercow Report: A review of services for children and young people (0–19) with speech, language and communication needs. UK: DCSF Publications; 2008.

[279] Cochran JC (2014). Breaches in the wall: Imprisonment, social support, and recidivism. J Res Crime Delinq.

[280] Hopkins K. The pre-custody employment, training and education status of newly sentenced prisoners. Results from the Surveying Prisoners Crime Reduction (SPCR) longitudinal cohort study of prisoners. 2012.

[281] Higgs T, Carter AJ (2015). Autism spectrum disorder and sexual offending: responsivity in forensic interventions. Aggress Violent Behav.

[282] Ray F, Marks C, Bray-Garretson H (2004). Challenges to treating adolescents with Asperger’s Syndrome who are sexually abusive. Sex Addict Compuls.

[283] Snow P, Powell M (2004). Interviewing juvenile offenders: the importance of oral language competence. Curr Issues Crim Justice.

[284] Joffe V (2011). Narrative Intervention Programme.

[285] Myers F (2004). On the borderline?: People with learning disabilities and/or autistic spectrum disorders in secure forensic and other specialist settings.

[286] Howes OD, Rogdaki M, Findon JL, Wichers RH, Charman T, King BH, Loth E, McAlonan GM, McCracken JT, Parr JR, Povey C (2018). Autism spectrum disorder: consensus guidelines on assessment, treatment and research from the British Association for Psychopharmacology. J Psychopharmacol.

[287] Bedrossian L (2015). Understand autism meltdowns and share strategies to minimize, manage occurrences. Disabil Compliance Higher Educ.

[288] National Collaborating Centre for Mental Health (UK). Autism: The Management and Support of Children and Young People on the Autism Spectrum. Leicester: Br Psychol Soc. 2013. 26065058

[289] Wong HH, Smith RG (2006). Patterns of complementary and alternative medical therapy use in children diagnosed with autism spectrum disorders. J Autism Dev Disord.

[290] Hanson E, Kalish LA, Bunce E, Curtis C, McDaniel S, Ware J, Petry J (2007). Use of complementary and alternative medicine among children diagnosed with autism spectrum disorder. J Autism Dev Disord.

[291] Moore ML, Eichner SF, Jones JR (2004). Treating functional impairment of autism with selective serotonin-reuptake inhibitors. Ann Pharmacother.

[292] Aman MG (2004). Management of hyperactivity and other acting-out problems in patients with autism spectrum disorder. Semin Pediatr Neurol..

[293] Arnold LE, Vitiello B, McDougle C, Scahill L, Shah B, Gonzalez NM, Chuang S, Davies M, Hollway J, Aman MG, Cronin P (2003). Parent-defined target symptoms respond to risperidone in RUPP autism study: customer approach to clinical trials. J Am Acad Child Adolesc Psychiatry.

[294] Findling RL, McNamara NK, Gracious BL, O'Riordan MA, Reed MD, Demeter C, Blumer JL (2004). Quetiapine in nine youths with autistic disorder. J Child Adolesc Psychopharmacol.

[295] Matson JL, Sipes M, Fodstad JC, Fitzgerald ME (2011). Issues in the management of challenging behaviours of adults with autism spectrum disorder. CNS Drugs.

[296] Kumar B, Prakash A, Sewal RK, Medhi B, Modi M (2012). Drug therapy in autism: a present and future perspective. Pharmacol Rep.

[297] Rotta K, VanDerwall R, Ehrhardt K, Poling A. Pharmacological Interventions for Adults with Autism Spectrum Disorder. In Handbook of Quality of Life for Individuals with Autism Spectrum Disorder. Cham: Springer International Publishing; 2022. pp. 293-310.

[298] Leaman J, Emslie L, Richards A, O’Moore E (2016). Rapid review of evidence of the impact on health outcomes of NHS commissioned health services for people in secure and detained settings to inform future health interventions and prioritisation in England.

[299] Adult Social Care: PSI 03.2016. April 2016. Accessed 3^rd^ Jan. https://www.gov.uk/government/publications/adult-social-care-psi-032016-pi-062016.

[300] Royal College of Psychiatrists (RcoP). Forensic care pathways for adults with intellectual disability involved with the criminal justice system. In.: Royal College of Psychiatrists’ Faculty of Psychiatry and Intellectual Disability and Faculty of Forensic Psychiatry. London: Royal College of Psychiatry. 2014.

[301] Care Programme Approach (CPA). Care for people with mental health problems (Care Programme Approach) – NHS (www.nhs.uk). Accessed 29^th^ Jan 2021.

[302] Georgiou M, Jethwa J (2021). Planning effective mental healthcare in prisons: findings from a national consultation on the Care Programme Approach in prisons. J Forens Leg Med.

[303] National Health Service (NHS) (Updated 16^th^ February 2018) Care for people with mental health problems (Care Programme Approach). https://www.nhs.uk/conditions/social-care-and-support-guide/help-from-social-services-and-charities/care-for-people-with-mental-health-problems-care-programme-approach/. Accessed 29^th^ Jan 2021.

[304] Milen MT, Nicholas DB (2017). Examining transitions from youth to adult services for young persons with autism. Soc Work Health Care.

[305] Enner S, Ahmad S, Morse AM, Kothare SV (2020). Autism: considerations for transitions of care into adulthood. Curr Opin Pediatr.

[306] Jarrett M, Thornicroft G, Forrester A, Harty M, Senior J, King C, Huckle S, Parrott J, Dunn G, Shaw J (2012). Continuity of care for recently released prisoners with mental illness: a pilot randomised controlled trial testing the feasibility of a critical time intervention. Epidemiol Psychiatr Sc.

[307] McDaid D, Park AL, Wahlbeck K (2019). The economic case for the prevention of mental illness. Ann Rev Public Health.

[308] Charman T (2011). The highs and lows of counting autism. Am J Psychiatry.

[309] Myles BS, Bock SJ, Simpson RL. ASDS: Asperger Syndrome Diagnostic Scale. Indianapolis: Pro-Ed; 2001.

[310] Ehlers S, Gillberg C, Wing L (1999). A screening questionnaire for Asperger syndrome and other high-functioning autism spectrum disorders in school age children. J Autism Dev Disord.

[311] Kopp S, Gillberg C (2011). The Autism Spectrum Screening Questionnaire (ASSQ)-Revised Extended Version (ASSQ-REV): An instrument for better capturing the autism phenotype in girls? A preliminary study involving 191 clinical cases and community controls. Res Dev Disabil.

[312] Schopler E, Reichler RJ, Renner BR (2010). The childhood autism rating scale (CARS).

[313] Gilliam JE (2001). Gilliam Asperger’s Disorder Scale: GADS.

[314] Gilliam JE (2014). GARS-3: Gilliam Autism Rating Scale-Third Edition (GARS-3).

[315] Krug DA. Krug asperger’s disorder index: Examiner’s manual. Indianapolis: Pro-ed; 2003.

[316] Derks O, Heinrich M, Brooks W, Sterkenburg P, McCarthy J, Underwood L, Sappok T (2017). The Social Communication Questionnaire for adults with intellectual disability: SCQ-AID. Autism Res.

